# Hepatitis B doubly spliced protein (HBDSP) promotes hepatocellular carcinoma cell apoptosis via ETS1/GATA2/YY1-mediated p53 transcription

**DOI:** 10.1128/jvi.01087-23

**Published:** 2023-11-06

**Authors:** Xiazhen Xu, Lu Zhang, Guiying Ye, Jiajian Shi, Yibin Peng, Fan Xin, Yi Lin, Qiong Wu, Xu Lin, Wannan Chen

**Affiliations:** 1 Key Laboratory of Gastrointestinal Cancer (Fujian Medical University), Ministry of Education, School of Basic Medical Sciences, Fujian Medical University, Fuzhou, China; 2 Fujian Key Laboratory of Tumor Microbiology, Department of Medical Microbiology, School of Basic Medical Sciences, Fujian Medical University, Fuzhou, China; University of Southern California, Los Angeles, California, USA

**Keywords:** hepatitis B virus, RNA splicing, *p53*, apoptosis, transactivator protein

## Abstract

Chronic hepatitis B virus (HBV) infection represents an important global public health concern. The spliced variants generated by RNA splicing from 3.5-kb HBV pre-genomic RNA are involved in chronic hepatitis B pathogenicity and associated with hepatocellular carcinoma development. Although the HBV spliced variants with a length of 2.2 kb have been widely detected, their roles in the development of HBV-associated liver diseases remain unknown. In the present study, the pro-apoptotic effects of hepatitis B doubly spliced protein (HBDSP) encoded by 2.2-kb doubly spliced variants of HBV in wild-type *p53* (wt*-p53*) hepatocytes were determined. We primarily found that HBDSP promoted the apoptosis of HepG2 and SMMC-7721 cells with wt*-p53*. The role of wt-*p53* in HBDSP-induced apoptosis was further investigated. It was demonstrated that HBDSP upregulated p53 and phospho-p53 (Ser15) expression and herein stimulated the *p53*-dependent apoptotic signaling pathway. Mechanistically, HBDSP indirectly transactivated the *p53* promoter in an ETS1-, GATA2-, and YY1-dependent manner, in which HBDSP increased the nuclear translocation of ETS1, GATA2, and YY1. Besides, it was demonstrated that HBDSP could promote cellular apoptosis and activate *p53*-dependent apoptotic signaling pathway, resulting in increased secretion of HBV DNA, HBsAg, and HBeAg in HepG2.2.15 cells and HBV-infected HepG2-NTCP cells. Taken together, our results reveal a novel mechanism by which HBDSP promotes ETS1/GATA2/YY1-dependent *p53* gene transcription and induces apoptosis in wt-*p53* cells, and increases the production of HBV progeny and viral antigens. These findings may provide novel insight into the pathogenesis of HBDSP involved in HBV-associated liver diseases.

**IMPORTANCE:**

Hepatitis B virus (HBV) spliced variants are associated with viral persistence or pathogenicity. Hepatitis B doubly spliced protein (HBDSP), which has been previously reported as a pleiotropic transactivator protein, can potentially serve as an HBV virulence factor. However, the underlying mechanisms of HBDSP in HBV-associated liver diseases remain to be elucidated. In this study, we revealed that HBDSP promotes cellular apoptosis and induces wt-*p53*-dependent apoptotic signaling pathway in wt-*p53* hepatocellular cells by transactivating p53 transcription, and increases the release of HBV progeny. Therefore, HBDSP may promote the HBV particles release through wt-*p53*-dependent hepatocellular apoptosis. Our findings suggest that blocking HBDSP-induced wt-*p53*-dependent apoptosis might have therapeutic values for chronic hepatitis B.

## INTRODUCTION

Globally, nearly 296 million individuals are living with chronic HBV infection, leading to a substantial global public health burden (1). Although hepatitis B vaccination has effectively decreased the rates of HBV infection among children in recent decades, more needs to be done to eliminate cirrhosis and liver cancer driven by chronic HBV infection (2). HBV is a partially double-stranded DNA virus that belongs to the hepadnaviridae family, four partially overlapped open reading frames constituting its 3.2-kb genome and encoding a core protein, DNA polymerase, surface envelope protein, and X protein (3). Additionally, the HBV genome also encodes some specific spliced proteins generated from RNA splicing and reverse transcription at specific sites from the 3.5-kb pre-genomic RNA (pgRNA) (4). Moreover, these spliced variants are closely related to persistent HBV infection and pathogenicity (5, 6). At least 17 species of HBV spliced variants have been identified in HBV cell cultures or the serums and liver tissues obtained from patients with chronic hepatitis B (CHB) (7). Of these, the 2.2-kb spliced variants of the HBV genome are the most common and can be divided into singly or doubly spliced variants, depending on the splicing pattern (8). The singly spliced variant (spliced between 2,447 nt and 489 nt) encodes hepatitis B spliced protein (HBSP) associated with liver fibrosis (9). HBSP could cause liver damage by inducing specific cytotoxic T lymphocyte (CTL) activity (10), and controversial research reported that the contribution of T-cell response to HBSP was probably weak with liver damage in the chronic HBV patients (11). The doubly spliced variant (spliced between 2,447 nt and 2,935 nt and between 3,018 nt and 489 nt) encodes HBDSP, which has transactivational activities on various oncogenes and HBV gene promoter sequences via AP-1- and C/EBP-binding sites. And the 48–75 amino acid residues of HBDSP were crucial for its transactivational activities (12). It has been well established that the 2.2-kb doubly HBV spliced variants increased the replication of wild-type HBV *in vitro* (13).

Viral hepatitis has been shown to be related to liver cell damage and cell death caused by viral infection (14). Apoptosis is a typical process responsible for cell death and exhibits prominent characteristics in the pathogenesis of liver chronic diseases (15). It has also been demonstrated that the *p53* gene is the most potent factor for inducing apoptosis (16). Moreover, *p53* is the most important tumor suppressor in mammalian genomes, as well as the most frequently mutated somatic gene in human cancers, which is both closely related to tumorigenesis, regulation of cell growth, differentiation, and death (17). The p53 protein is a tightly regulated transcription factor responsible for maintaining the integrity of cellular DNA under various stimuli (e.g., ionizing radiation and oxidative stress), thereby functioning as a tumor suppressor (18). Wt-p53, when phosphorylated as p-p53, can trigger cell death through both apoptosis and ferroptosis (19). Phosphorylation of p53 at multiple sites can modulate different physiological functions. For instance, phosphorylation of the amino-terminal serine 15 of p53, denoted as p-p53 (Ser15), has been documented to enhance p53 activation, increase p53 stability and pro-apoptotic activity, and promote the accumulation of p53 within cells (20). The accumulation of intracellular-activated p53 protein resulted in the transcriptional transactivation of pro-apoptotic genes (e.g., Bax and Fas) or inhibition of anti-apoptotic genes (e.g., Bcl-2). These changes in gene expression induced mitochondrial apoptosis or death receptor pathway of apoptosis and inhibited cellular proliferation (21, 22). Additionally, it has been observed that hepatitis B virus X protein (HBx) overexpression in hepatocellular carcinoma (HCC) cells elevates p53 phosphorylation at Ser15 and upregulates *p53-*dependent target genes, thereby facilitating cellular apoptosis (23). Moreover, it was shown that the wt*-*p53 could suppress proliferation and stimulate apoptosis in hepatoma cells (24). However, the mutant *p53* (mut-*p53*) gene resulted in a loss of wt*-p53* gene activity, leading to inactivation of the p53 signaling pathway and apoptosis inhibition in HCC (25, 26).

It has been previously demonstrated that HBSP proteins can induce Huh7 cell apoptosis without blocking the cell cycle and exacerbating hepatitis (6). In the present study, we primarily found that HBDSP promoted apoptosis in the wt-*p53* HepG2 and SMMC-7721 cell strains but not in mut-*p53* Huh7 and MHCC-97H cell strains by flow cytometry. The pro-apoptotic effects of HBDSP were confirmed to be associated with *p53*, and the *p53*-mediated apoptotic signaling pathway was identified using the p53 inhibitor, pifithrin-α (PFTα), and p53 short interfering RNA (siRNA). Furthermore, it was demonstrated that HBDSP could transactivate the *p53* promoter, and the binding sites of transcriptional factors ETS1, GATA2, and YY1 in the −595 nt to −360 nt regions of the *p53* promoter were essential for HBDSP-regulated wt-*p53*-mediated apoptosis. Interestingly, the *p53* promoter was activated by HBDSP via inducing the nuclear translocation of ETS1, GATA2, and YY1, rather than affecting the levels of ETS1, GATA2, and YY1 protein expression. Our findings also confirmed the absence of protein-protein interactions between HBDSP and ETS1, GATA2, or YY1, together with the absence of interactions between HBDSP and *p53* promoter. Additionally, HBDSP was demonstrated to induce cellular apoptosis and activate the *p53*-mediated apoptotic signaling pathway in both HepG2.2.15 cells containing HBV genomic DNA and HepG2-NTCP cells infected with HBV. Meanwhile, HBDSP also increased the release of HBV DNA, HBsAg (HBV surface antigen), and HBeAg (HBV e antigen) into the culture supernatants of HepG2.2.15 cells and HBV-infected HepG2-NTCP cells.

## RESULTS

### HBDSP promotes hepatocellular apoptosis in HepG2 and SMMC-7721 cells

**Fig 1:**
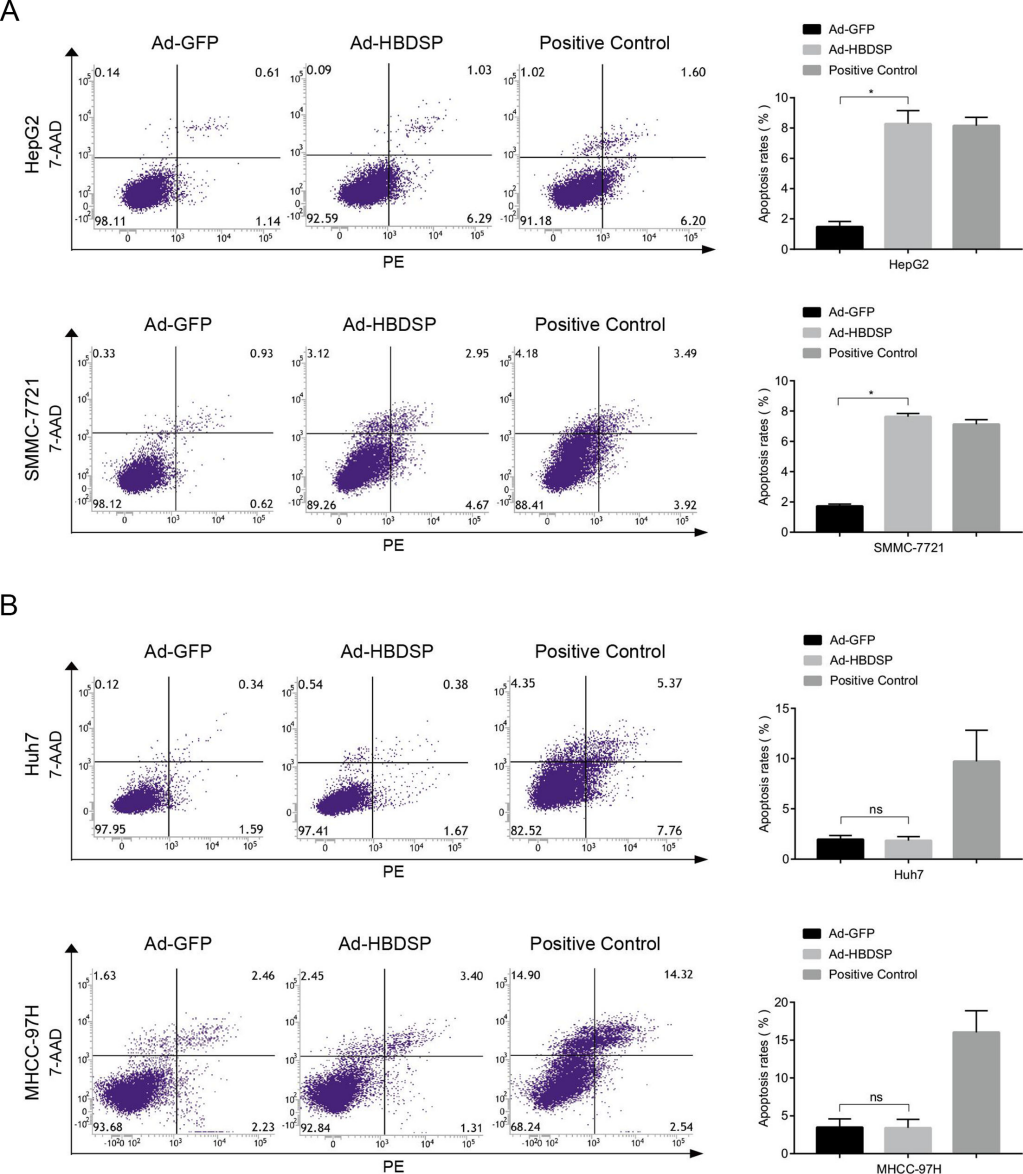
HBDSP-induced hepatocellular apoptosis in HepG2 and SMMC-7721 cells. (**A and B**) Apoptosis assay. HepG2, SMMC-7721, Huh7, and MHCC-97H cell lines were, respectively, infected with the Ad-HBDSP or Ad-GFP at multiplicities of infection of 20, 50, 60, or 100 for 72 h followed by an analysis using a PE Annexin-V staining and flow cytometry. The ratios of apoptosis in HBDSP-infected HepG2 and SMMC-7721 cells were, respectively, upregulated by 460.50% and 344.27%, compared with the GFP-infected control, whereas the Huh7 and MHCC-97H cell lines displayed no significant changes. The group induced by the Apoptosis Inducers Kit was used as a positive control. All assays were performed in triplicate. Data are presented as the means ± SD. *P < 0.05 compared to the control. ns, non-significant.

To investigate the effects of HBDSP on cell apoptosis, HBDSP was overexpressed by recombinant adenovirus infection in the human hepatoblastoma cell line, HepG2, and three human hepatocellular carcinoma cell lines, SMMC-7721, Huh7, and MHCC-97H (Fig. S1). Next, the effects of HBDSP overexpression on cell apoptosis were detected by flow cytometry. The results revealed that HBDSP could significantly induce apoptosis in HepG2 and SMMC-7721 cells (Fig. 1A) but not in Huh7 and MHCC-97H cells (Fig. 1B). The apoptotic assay was repeated in HepG2, SMMC-7721, Huh7, and MHCC-97H cells transfected with phouge-HBDSP plasmids (Fig. S2) to obtain the similar results.

### *p53* is involved in HBDSP-induced apoptosis

**Fig 2:**
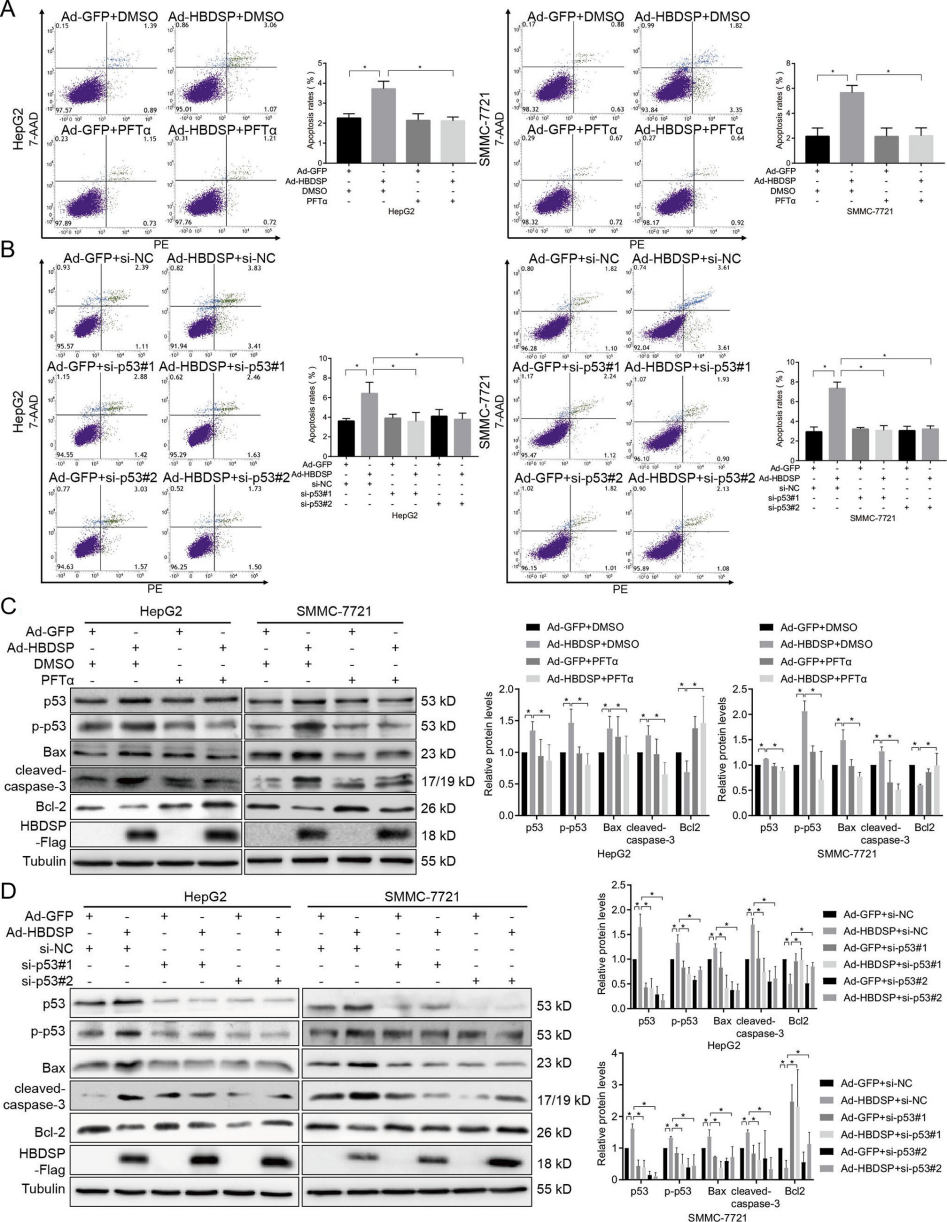
p53 was implicated in HBDSP-induced apoptosis. (**A**) Apoptosis assay following PFTα treatment. HepG2 and SMMC-7721 cell lines were, respectively, treated with 20 µM PFTα or DMSO for 6 h following infection with Ad-HBDSP or Ad-GFP at various multiplicities of infection (MOIs) for 66 h followed by an analysis using PE Annexin-V staining and flow cytometry. The apoptosis ratios in HBDSP-infected HepG2 and SMMC-7721 cells were, respectively, upregulated by 65.09% and 164.33%, compared with the GFP-infected control groups treated with DMSO. The apoptosis ratios were reversed in the PFTα-treated groups compared with the DMSO-treated groups in the case of HBDSP overexpression. (**B**) Apoptosis assay following siRNA treatment. HepG2 and SMMC-7721 cell lines were, respectively, transfected with 50 nM of p53 siRNA (si-p53#1 and si-p53#2) or negative control siRNA (si-NC) for 24 h after infection with Ad-HBDSP or Ad-GFP at various MOIs for 48 h followed by an analysis using a PE Annexin-V staining and flow cytometry. The apoptosis ratios in HBDSP-infected HepG2 and SMMC-7721 cells were, respectively, upregulated by 78.00% and 150.97%, compared with the GFP-infected control under si-NC treatment. The apoptosis ratios were recovered in the si-p53#1- or si-p53#2-transfected groups compared with the si-NC-transfected groups in the case of HBDSP overexpression. (**C**) Western blot analysis of the *p53-*mediated apoptosis signaling pathway. HepG2 and SMMC-7721 cell lines were, respectively, treated with 20 µM PFTα or DMSO for 6 h following infection with Ad-HBDSP or Ad-GFP at various MOIs for 66 h, and the levels of p53, p-p53 (Ser15), Bax, cleaved-caspase-3, and Bcl-2 protein expression were detected by Western blot analysis. (**D**) Western blot analysis of the *p53-*mediated apoptosis signaling pathway. HepG2 and SMMC-7721 cell lines were, respectively, transfected with 50 nM of p53 siRNA or NC siRNA for 24 h after infection with Ad-HBDSP or Ad-GFP at various MOIs for 48 h, and the levels of p53, p-p53 (Ser15), Bax, cleaved-caspase-3, and Bcl-2 protein expression were detected by Western blot analysis. Relative protein quantitative values were normalized to the corresponding internal control tubulin. All assays were performed in triplicate. Data are shown as the means ± SD. *P < 0.05 compared to the control.

Previous studies have demonstrated that HepG2 and SMMC-7721 cells were wt-*p53* cell lines, whereas Huh7 and MHCC-97H were mut-*p53* cell lines (27, 28). We postulated that the differential effects of HBDSP on the induction of hepatocellular apoptosis might be associated with the *p53* gene. Therefore, to examine the roles of wt-*p53* in HBDSP-induced apoptosis, the p53-specific inhibitor, PFTα, and p53-specific siRNA were used to inhibit p53 expression in HepG2 and SMMC-7721 cells. The flow cytometry results revealed that the apoptotic rates were increased by HBDSP induction but significantly downregulated following treatment with 20 µM PFTα compared with the dimethyl sulfoxide (DMSO)-treated control (Fig. 2A). Moreover, when p53 siRNA (si-p53#1 and si-p53#2) were used to decrease p53 expression, the apoptotic rates were also reduced compared with the si-NC control (Fig. 2B). These results demonstrate that hepatocellular apoptosis induced by HBDSP was associated with *p53*. Furthermore, the results in Fig. 2C and D revealed that HBDSP overexpression increased the expression levels of p53, p-p53 (Ser15), p53-signaling pathway-related Bax, and cleaved-caspase-3 but decreased the expression levels of Bcl-2. However, levels of p-p53 (Ser15), Bax, and cleaved-caspase-3 protein expression were downregulated, and Bcl-2 was upregulated by using PFTα and p53 siRNA to inhibit the p53 expression. These data demonstrate that HBDSP promotes apoptosis via activating the *p53-*mediated apoptotic signaling pathway.

Our previous study identified that HBDSP has transactivational activities, and the residues Leu48 and Gln75 were the crucial transactivating domains (12). To elucidate the role of the 48–75 residues of HBDSP in HBDSP-induced apoptosis, the effects of HBDSPΔ48–75 on apoptosis in HepG2 and SMMC-7721 cells were investigated also. The results depicted in Fig. S3 revealed that no significant change in the apoptotic rate was observed in the HBDSPΔ48–75 mutant compared to the control group.

### HBDSP transcriptionally transactivates the *p53* promoter

**Fig 3:**
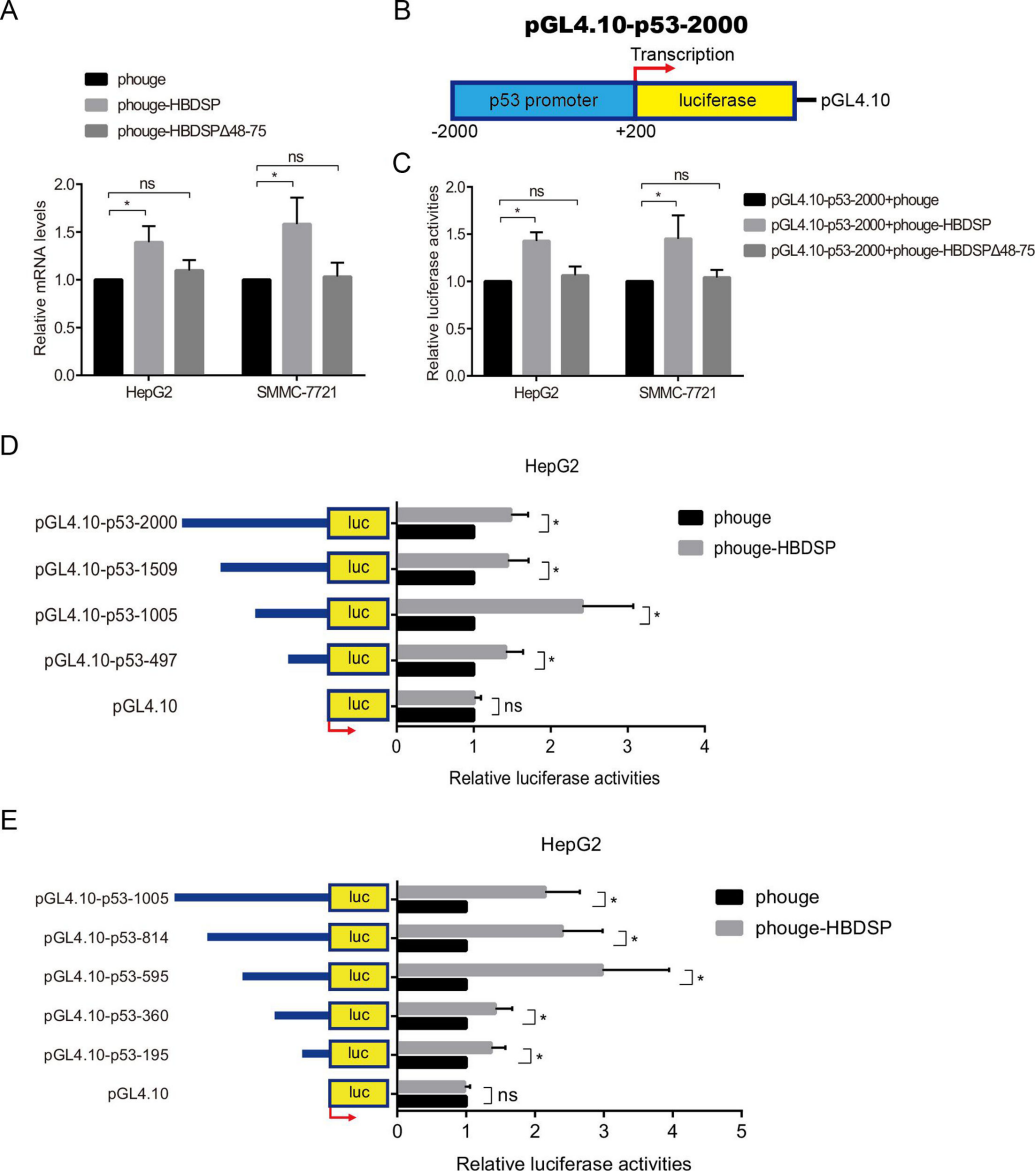
HBDSP regulated the −595 nt to −360 nt regions of the *p53* promoter via transactivational activities. (**A**) qRT-PCR for detecting the levels of *p53* mRNA expression. HepG2 and SMMC-7721 cell lines were transfected with phouge-HBDSP, phouge-HBDSPΔ48–75, or control empty plasmids for 48 h followed by an analysis using a qRT-PCR assay. The relative mRNA quantitative values of *p53* were normalized to the corresponding GAPDH internal control. The groups transfected with empty plasmids were set to 1. (**B**) Schematic of the *p53-*promoter reporter vector (pGL4.10-p53-2000). (**C**) Luciferase reporter assay detected the *p53* promoter activities. HepG2 and SMMC-7721 cell lines were, respectively, co-transfected with phouge-HBDSP, phouge-HBDSPΔ48–75, or phouge and pGL4.10-p53-2000 plasmids for 48 h, and luciferase activities were measured using a luciferase reporter assay. The empty phouge vector transfection groups served as the control, which was set to 1. (**D and E**) Luciferase reporter assay for detecting the activities of a series of *p53* promoter constructs with 5′-deletion. HepG2 cells were co-transfected with phouge-HBDSP or phouge and a series of *p53* promoter constructs with 5′-deletion for 48 h, and the luciferase activities were analyzed. The empty phouge vector transfection groups were set to 1. All assays were performed in triplicate. Data are presented as the means ± SD. *P < 0.05 compared to control. ns, non-significant.

To further explore the mechanisms by which HBDSP induces the upregulation of p53 expression, the levels of *p53* transcription in HepG2 and SMMC-7721 cells were examined. Real-time quantitative reverse transcription PCR (qRT-PCR) results indicated increased levels of *p53* mRNA following HBDSP overexpression, while there was no significant change of *p53* mRNA levels following the overexpression of HBDSP∆48–75 deletion construct (Fig. 3A). Moreover, we further evaluated the transactivational effects of HBDSP on the *p53* promoter. The pGL4.10-p53-2000 plasmids harboring the full length of the *p53* promoter (nucleotides −2,000 to +200) (Fig. 3B) were constructed using pGL4.10 as an empty vector. HepG2 and SMMC-7721 cells were, respectively, co-transfected with phouge-HBDSP, phouge-HBDSP∆48–75, or phouge and pGL4.10-p53-2000 plasmids, and the activities of the *p53* promoter were measured with an equal amount of each sample. The luciferase reporter assay revealed that HBDSP activated the *p53* promoter, resulting in increased transcription of the *p53* gene. In contrast, HBDSP∆48–75 deletion construct did not activate the *p53* promoter compared to the empty control, which was set to 1 (Fig. 3C).

### −595 nt to −360 nt regions of the *p53* promoter are crucial for HBDSP transactivational regulation

To determine the functional regions by which HBDSP regulates the *p53* promoter, a series of *p53* promoter constructs with 5′-deletion were constructed (pGL4.10-p53-1509, nucleotides −1,509 to +200; pGL4.10-p53-1005, nucleotides −1,005 to +200; pGL4.10-p53-497, nucleotides −497 to +200). Next, the phouge-HBDSP plasmids were, respectively, co-transfected with pGL4.10-p53-2000, pGL4.10-p53-1509, pGL4.10-p53-1005, and pGL4.10-p53-497 into HepG2 cells. As shown in Fig. 3D, in each *p53* promoter subclones, the relative luciferase activities of pGL4.10-p53-2000, pGL4.10-p53-1509, pGL4.10-p53-1005, and pGL4.10-p53-497 were increased by HBDSP overexpression compared with the control group transfected with phouge, which was set to 1. Importantly, the relative luciferase activities of pGL4.10-p53-1005 were significantly higher than the other constructs. Then, to further determine the most critical regions through which HBDSP regulates the *p53* promoter, a series of approximately 200 nt 5′-deletions in nucleotides −1,005 to +200 were subcloned into the pGL4.10 vector. The phouge-HBDSP and pGL4.10-p53-1005, pGL4.10-p53-814 (nucleotides −814 to +200), pGL4.10-p53-595 (nucleotides −595 to +200), pGL4.10-p53-360 (nucleotides −360 to +200), and pGL4.10-p53-195 (nucleotides −195 to +200) were transiently co-transfected into HepG2 cells, respectively. The results in Fig. 3E exhibited that the relative luciferase activities of pGL4.10-p53-1005, pGL4.10-p53-814, pGL4.10-p53-595, pGL4.10-p53-360, and pGL4.10-p53-195 were higher by HBDSP overexpression than the control group transfected with empty vector. Moreover, the pGL4.10-p53-595 plasmid exhibited the maximum luciferase activities compared with the other *p53* promoter region constructs. These findings suggest that nucleotides in the −595 nt to −360 nt regions may be essential for the full activities of the *p53* promoter.

### ETS1, GATA2, and YY1 bind to the −595 nt to −360 nt regions of the *p53* promoter both *in vitro* and *in vivo*

**Fig 4:**
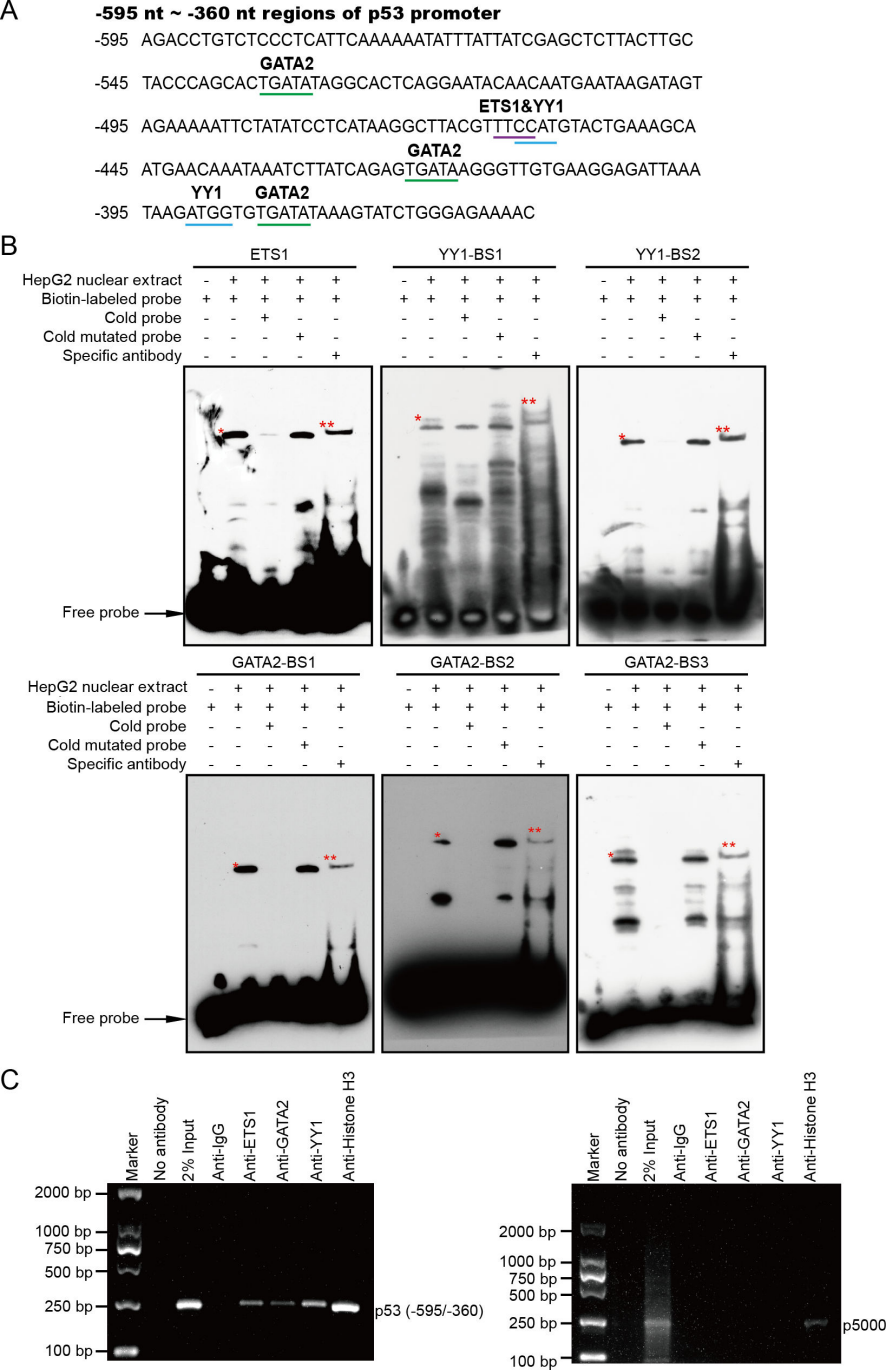
ETS1, GATA2, and YY1 could bind to the −595 nt to −360 nt regions of the *p53* promoter both *in vitro* and *in vivo*. (**A**) Nucleotide sequences of the −595 nt to −360 nt regions of the *p53* promoter. PROMO, JASPAR, and Gene Regulation databases were used to predict putative transcription factors and their binding sites. Three putative GATA2-binding sites, one ETS1-binding site, and two YY1-binding sites, were underlined in the −595 nt to −360 nt regions of the *p53* promoter. (**B**) Electrophoretic mobility shift assay and a super-shift assay of ETS1, GATA2, and YY1 binding to the *p53* promoter *in vitro*. The 5′-biotin end-labeled probes were incubated in the absence (lane 1) or presence (lane 2) of nuclear extracts from HepG2 cells. The unlabeled cold probes (lane 3) and unlabeled cold mutated probes (lane 4) were used as competitors at a concentration of a 100-fold molar excess to the biotin-labeled probes. Super-shift assays were performed using 10 µg of specific antibodies against ETS1, GATA2, or YY1 (lane 5). The specific protein-DNA complexes (shown as the shift band) were indicated using one asterisk, and the super-shift bands were marked by two asterisks. BS, binding site; * shift band; ** super-shift band. (**C**) The chromatin immunoprecipitation assay of ETS1, GATA2, and YY1 binding to the *p53* promoter *in vivo*. Chromatin from HepG2 cells was immunoprecipitated with the anti-ETS1, -GATA2, or -YY1 antibodies. The total extracted DNA (2% Input) prior to immunoprecipitation, as well as the immunoprecipitated and purified DNA were amplified by PCR using specific primers for the regions that spanned −595 nt to −360 nt (containing the ETS1-, GATA2-, and YY1-binding sites) of the *p53* promoter. Pre-blocked protein A/G or normal rabbit IgG or Histone H3 antibodies were used as controls. p5000 primers were used as a negative control for *p53*.

We speculated that the −595 nt to −360 nt regions of the *p53* promoter may be HBDSP-regulated cis-acting elements. To further determine the specific mechanisms of HBDSP-mediated regulation of the *p53* promoter, PROMO, JASPAR, and Gene Regulation databases were, respectively, used to predict the putative transcription factors and their binding sites in the nucleotides −595 nt to −360 nt regions of the *p53* promoter. Subsequently, three putative transcription factors, ETS1, GATA2, and YY1, together with six binding sites for these transcription factors, were screened from the intersection of predicted transcription factors by the three databases (Fig. 4A).

To confirm the binding of ETS1, GATA2, and YY1 to the *p53* promoter in HepG2 cells, nuclear extracts from HepG2 cells were subjected to an electrophoretic mobility shift assay (EMSA) and super-shift assay using specific antibodies against ETS1, GATA2, or YY1. The results in Fig. 4B showed the binding of the complexes of ETS1, GATA2, or YY1 with the *p53* promoter. Shift bands (the slow migration bands indicated in lane 2) suggested that the complexes were formed by the combination of the 5′-biotin end-labeled probes of ETS1, GATA2, or YY1 and the nuclear extracts. The addition of a 100-fold molar excess of unlabeled cold probes reduced the complex (lane 3). In contrast, the 100-fold molar excess of unlabeled cold mutated probes attenuated this reduction, and the complexes reappeared (lane 4). Super-shift bands appeared when the nuclear extracts were pre-incubated with 5′-biotin end-labeled probes and ETS1, GATA2, or YY1 antibodies (indicated as another slower-migration bands in lane 5). These results confirm that ETS1, GATA2, and YY1 can specifically bind to the *p53* promoter *in vitro*.

A chromatin immunoprecipitation (ChIP) assay was also performed to further confirm the binding of ETS1, GATA2, and YY1 with the *p53* promoter *in vivo*. Anti-ETS1, -GATA2, or -YY1 antibodies or normal rabbit IgG or anti-histone H3 antibodies and chromatin extracted from HepG2 cells were used. Following immunoprecipitation, the DNA was purified and detected by PCR with specific primers for the region containing the ETS1-, GATA2-, and YY1-binding sites. As shown in Fig. 4A and C, a 235-bp DNA fragment containing the ETS1-, GATA2-, and YY1-binding sites was amplified, and the same band was observed in the groups of input DNA and anti-histone H3. No bands were detected in the control groups without antibodies and normal IgG. In addition, the negative control primers were designed at −5,000 nt of the *p53* promoter. There were no bands with groups of normal IgG, anti-ETS1, anti-GATA2, and anti-YY1; however, bands were detected with the groups of input DNA and anti-histone H3. In addition, a qPCR assay was performed with the purified DNA products to quantify them by using specific primers targeting the −595 nt/−360 nt region of the *p53* promoter. The results in Fig. S4A showed that *p53* promoter DNA sequences were enriched by ETS1, GATA2, and YY1, which was consistent with the Fig. 4C results. Taken together, these results confirm that ETS1, GATA2, and YY1 can directly bind to the *p53* promoter *in vivo*, which are in-line with the *in vitro* EMSA results.

### HBDSP promotes the binding of ETS1, GATA2, and YY1 transcription factors to the *p53* promoter

**Fig 5:**
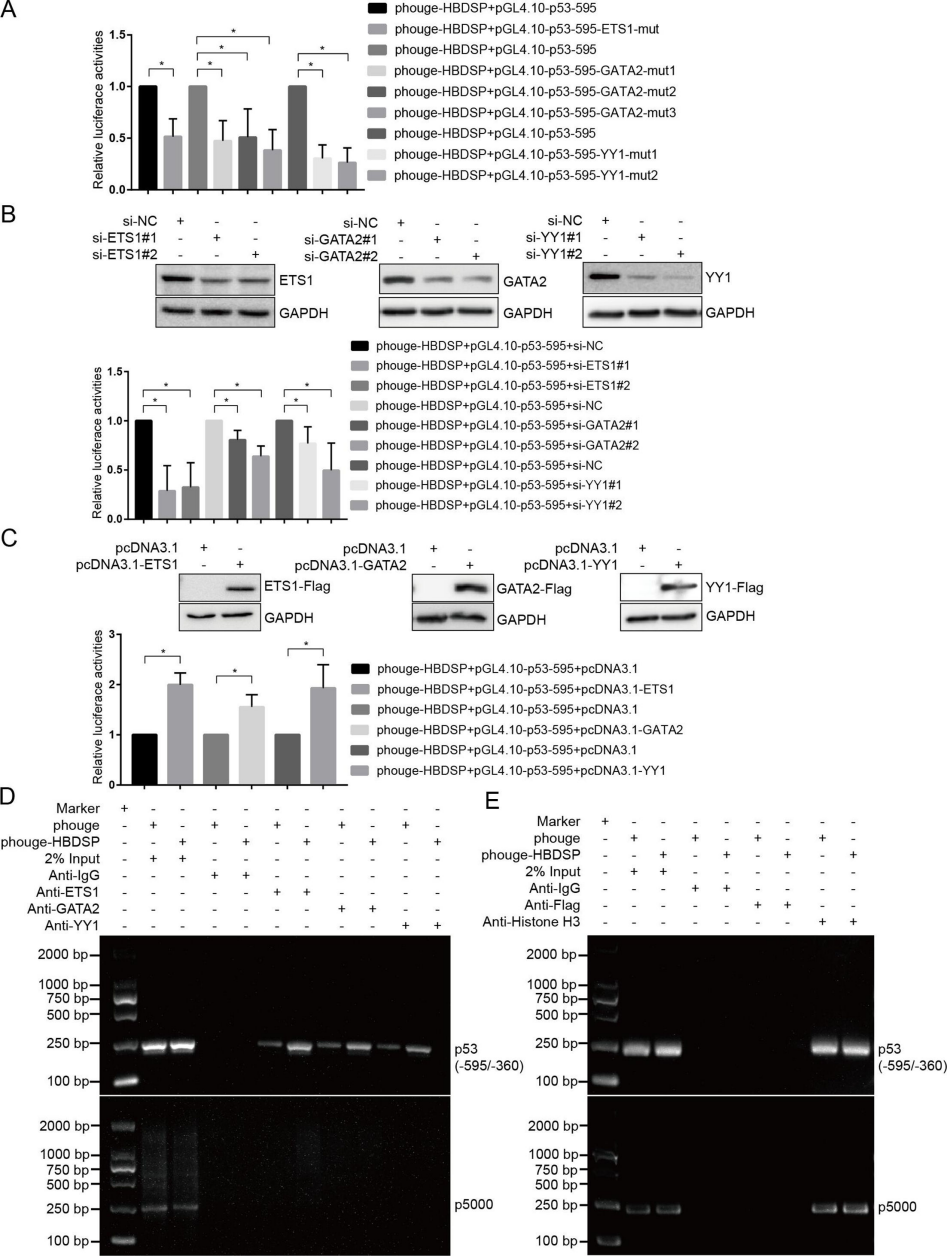
HBDSP enhanced the binding of transcription factors ETS1, GATA2, and YY1 to *p53* promoter. (**A**) Luciferase reporter assay detecting the effects of ETS1-, GATA2-, or YY1-binding site mutations in the *p53* promoter. ETS1-, GATA2-, or YY1-binding sites were subjected to site-directed mutagenesis. Phouge-HBDSP and pGL4.10-p53-595 or mutant constructs of the ETS1-, GATA2-, or YY1-binding sites were co-transfected into HepG2 cells, and the luciferase activities were detected at 48 h after transfection. The relative luciferase activities were obtained by comparison with the wild-type pGL4.10-p53-595, which was set to 1. (**B**) A luciferase reporter assay was performed to detect the effects of knocking down endogenous ETS1, GATA2, or YY1 on *p53* promoter activities. ETS1, GATA2, or YY1 siRNA were, respectively, transfected into HepG2 cells. Cells were harvested 48 h post-transfection and detected by Western blot using anti-ETS1, -GATA2, or -YY1 antibodies. HepG2 cells were co-transfected with phouge-HBDSP, pGL4.10-p53-595, and ETS1, GATA2, or YY1 siRNA, and the luciferase activities were detected at 48 h. The relative luciferase activities were obtained by a comparison with the si-NC, which was set to 1. (**C**) A luciferase reporter assay was performed to detect the effects of ETS1, GATA2, or YY1 overexpression on *p53* promoter activities. ETS1, GATA2, or YY1 constructs were, respectively, transfected into HepG2 cells. Cells were harvested 48 h after transfection and detected by Western blot using anti-ETS1, -GATA2, or -YY1 antibodies. HepG2 cells were co-transfected with phouge-HBDSP, pGL4.10-p53-595, and ETS1, GATA2, or YY1 constructs, and the luciferase activities were detected at 48 h after transfection. The relative luciferase activities were obtained by a comparison with the empty pcDNA3.1, which was set to 1. All assays were performed in triplicate. Data were shown as the means ± SD. *P < 0.05 compared to the control. (**D and E**) ChIP assay of ETS1, GATA2, and YY1 binding to the *p53* promoter in HBDSP-transfected HepG2 cells. Chromatin from HepG2 cells transfected with or without phouge-HBDSP was immunoprecipitated with the anti-ETS1, -GATA2, -YY1, or -Flag antibodies. The total extracted DNA (2% input) prior to immunoprecipitation, as well as the immunoprecipitated and purified DNA were amplified by PCR. Normal rabbit IgG or histone H3 antibodies were used as controls. p5000 primers were used as a negative control for *p53*.

To validate HBDSP-mediated regulation of *p53* via promoting the binding of ETS1, GATA2, and YY1 to the promoter, the mutants of six binding sites corresponding to three transcription factors were constructed using pGL4.10-p53-595 as a template. The phouge-HBDSP plasmids were, respectively, co-transfected with pGL4.10-p53-595 and six mutants (pGL4.10-p53-595-ETS1-mut; pGL4.10-p53-595-GATA2-mut1, -mut2, and -mut3; and pGL4.10-p53-595-YY1-mut1 and -mut2) into HepG2 cells, and the promoter activities were detected by a luciferase reporter assay. The results showed that the promoter activities of the mutated constructs were significantly lower than those of the non-mutated pGL4.10-p53-595 (Fig. 5A). The results suggest that HBDSP might regulate the *p53* promoter through regulating the binding of ETS1, GATA2, and YY1 to the *p53* promoter. Moreover, the phouge-HBDSP plasmids were, respectively, co-transfected with pGL4.10-p53-595 and ETS1-, GATA2-, and YY1-specific siRNA into HepG2 cells. The results in Fig. 5B indicated that in the HBDSP-transfected HepG2 cells, ETS1 knock down by si-ETS1#1 and #2 or GATA2 knock down by si-GATA2#1 and #2 or YY1 knock down by si-YY1#1 and #2 led to significant reductions in the promoter activities compared with the si-NC control. Furthermore, recombinant plasmids expressing ETS1, GATA2, and YY1 were constructed, and HepG2 cells were, respectively, co-transfected with the phouge-HBDSP and pGL4.10-p53-595. The results revealed that the promoter activities of pGL4.10-p53-595 were enhanced by HBDSP following the overexpression of ETS1, GATA2, and YY1 compared with the empty vector control (Fig. 5C). An *in vivo* ChIP assay was performed using HepG2 cell extracts transfected with or without phouge-HBDSP. As shown in Fig. 5D, the corresponding *p53* promoter regions were amplified following immunoprecipitation with antibodies against ETS1, GATA2, or YY1. When HBDSP was overexpressed in the cells, the intensity of the amplified p53 fragments was increased, suggesting that HBDSP expression may enhance the amounts of nuclear ETS1, GATA2, and YY1, thereby promoting their binding with the *p53* promoter. To elucidate whether HBDSP could directly bind to *p53* promoter, ChIP assay was subjected, and antibodies against Flag were used to immunoprecipitate HBDSP. The results in Fig. 5E showed that 235-bp amplified DNA fragments were observed in the groups of input DNA and anti-histone H3, but no bands were detected in the groups of anti-Flag and normal IgG, which suggested that HBDSP could not directly bind to *p53* promoter.

And the qPCR assays were also used to quantify the purified DNA products. Consistently, HBDSP could increase the enrichment of *p53* promoter DNA by transcription factors ETS1, GATA2, and YY1 as well (Fig. S4B). Furthermore, an *in vitro* EMSA was also performed using nuclear extracts from HepG2 cells transfected with phouge-HBDSP. As shown in Fig. S5, ETS1, GATA2, and YY1 could also specifically bind to the *p53* promoter *in vitro* upon HBDSP overexpression. Collectively, these results demonstrate that HBDSP plays a prominent role in promoting the binding of ETS1, GATA2, and YY1 to the *p53* promoter.

### HBDSP promotes the nuclear translocation of ETS1, GATA2, and YY1

**Fig 6:**
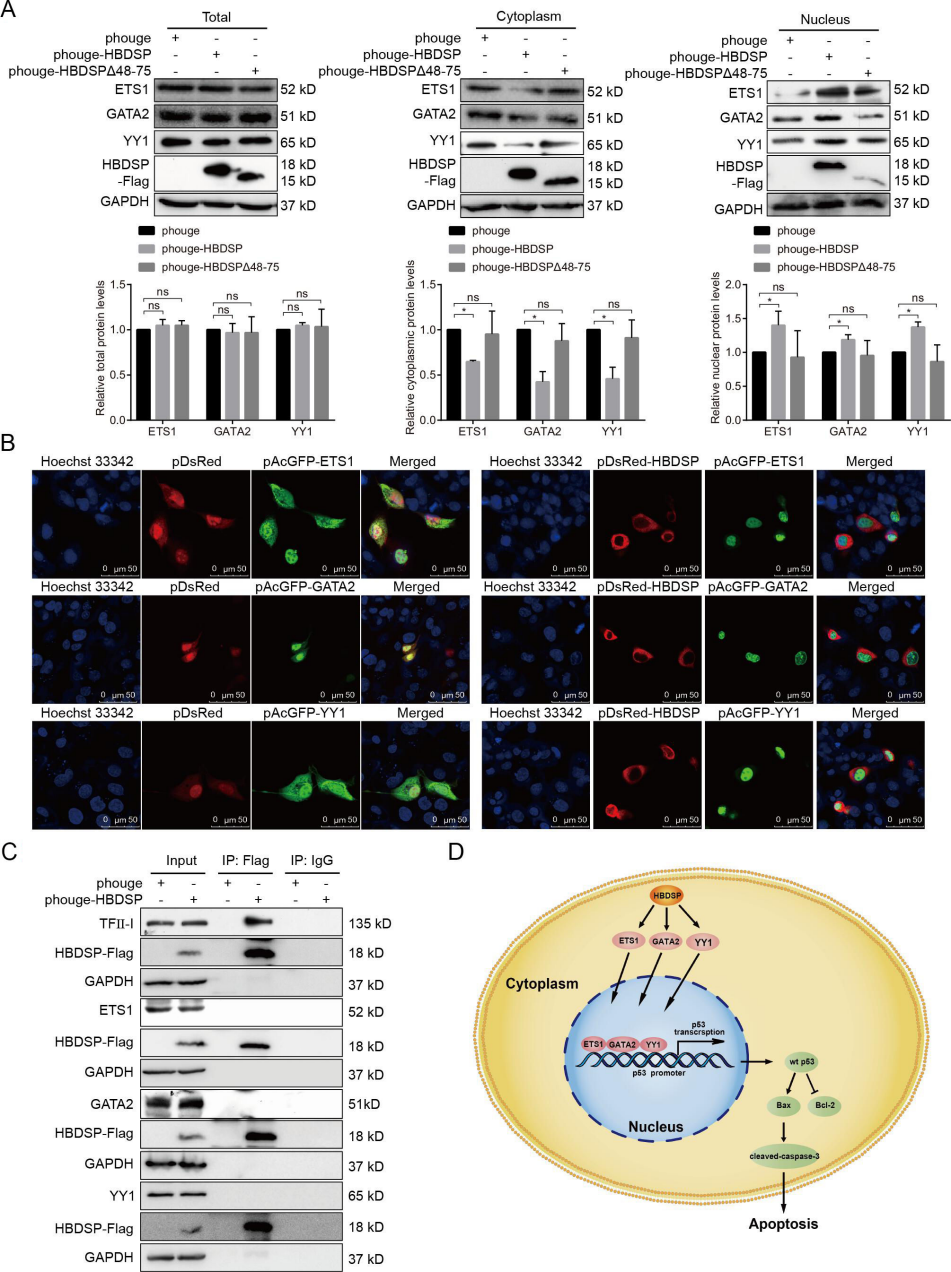
HBDSP promoted the nuclear translocation of ETS1, GATA2, and YY1. (**A**) Western blot analysis. HepG2 cells were transfected with phouge-HBDSP, phouge-HBDSPΔ48–75, or phouge for 48 h, and the levels of ETS1, GATA2, and YY1 expression in the total cellular, cytoplasmic, or nuclear proteins were detected. Proteins were probed with anti-ETS1, -GATA2, -YY1, or -Flag antibodies, and anti-GAPDH, or -histone H3 antibodies served as the loading control. The relative levels of proteins expression were obtained by a comparison with the empty phouge plasmids, which was set to 1. All assays were performed in triplicate. Data are shown as the means ± SD. *P < 0.05 compared to the control. ns, non-significant. (**B**) Confocal microscopy assay. HepG2 cells were co-transfected with pDsRed-HBDSP or pDsRed and pAcGFP-ETS1, -GATA2, or -YY1 for 48 h, followed by an incubation with Hoechst 33342 to stain the nucleus. (**C**) Co-immunoprecipitation assay. HepG2 cells were co-transfected with phouge-HBDSP or a phouge empty vector for 48 h, and immunoprecipitated lysates were detected with anti-ETS1, -GATA2, -YY1, -TFII-I, or -Flag antibodies by a Western blot analysis. GAPDH served as a loading control, and TFII-I served as a positive control. The normal rabbit IgG was used as a negative control. (**D**) Model of postulated mechanisms of HBDSP-induced apoptosis via p53 by promoting the nuclear translocation of ETS1, GATA2, and YY1.

To further investigate the mechanisms by which HBDSP promotes the binding of ETS1, GATA2, and YY1 to the *p53* promoter, the effects of HBDSP on the levels of ETS1, GATA2, and YY1 total protein expression were first detected. As indicated in Fig. 6A, HBDSP did not affect the levels of ETS1, GATA2, and YY1 protein expression in HepG2 cells. Next, both cytoplasmic and nuclear proteins were isolated to evaluate the effects of HBDSP on ETS1, GATA2, and YY1 expression in the cytoplasm and nucleus. The results of Western blot demonstrated that with HBDSP expression, the levels of ETS1, GATA2, and YY1 in the cytoplasm decreased notably, whereas ETS1, GATA2, and YY1 in the nucleus were significantly increased (Fig. 6A, lane 2 vs lane 1). However, the overexpression of HBDSPΔ48–75 did not affect the expression levels of ETS1, GATA2, and YY1 in the cytoplasm and nucleus compared with the empty vector control (Fig. 6A, lane 3 vs lane 1).

Consistently, the pDsRed-tagged HBDSP plasmids were, respectively, co-transfected with green fluorescent protein (GFP)-tagged ETS1, GATA2, or YY1, and detected using a confocal microscopy assay. As shown in Fig. 6B, the GFP-tagged ETS1, GATA2, or YY1 was primarily distributed in the nucleus in the HBDSP-expressed group, whereas they were both distributed in the cytoplasm and nucleus of the control group. Furthermore, a co-immunoprecipitation assay was performed to exclude the direct interaction of HBDSP with ETS1, GATA2, or YY1. The results in Fig. 6C showed an absence of protein-protein interactions between HBDSP and ETS1, GATA2, or YY1. Taken together, these data suggest that HBDSP can promote the nuclear translocation of ETS1, GATA2, and YY1 to bind with the *p53* promoter and facilitate *p53* transcription.

### HBDSP promotes HBV progeny by activating the *p53-*mediated apoptotic signaling pathway both in HBV-replicating and HBV-infected cells

**Fig 7:**
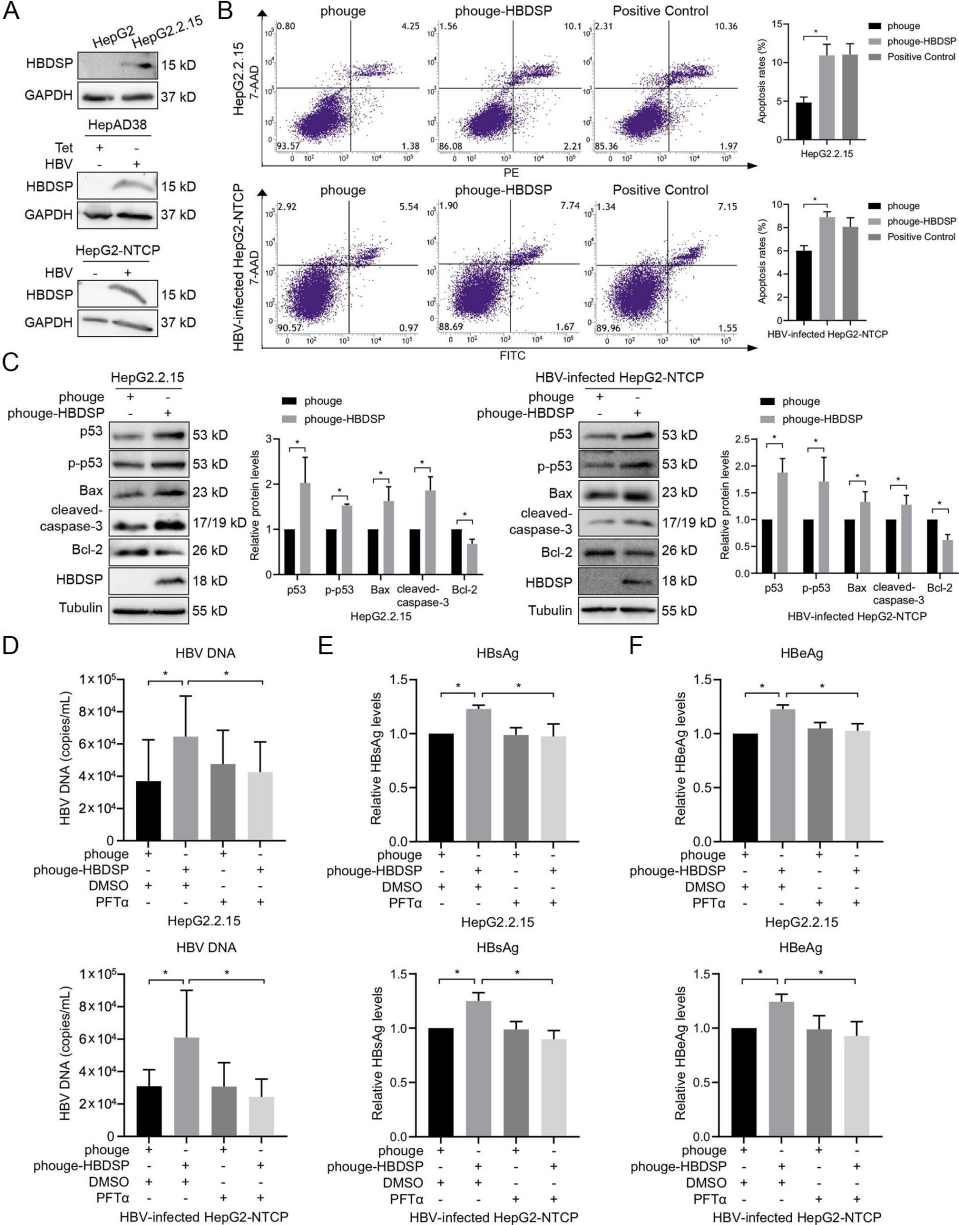
HBDSP regulated the *p53*-mediated apoptotic signaling pathway and promoted the HBV progeny. (**A**) Western blot analysis. The levels of HBDSP were detected in the HepG2 and HepG2.2.15 cells, in the HepAD38 cells treated with tetracycline (Tet) or not, and in the HepG2-NTCP cells infected with HBV or not. Proteins were probed with anti-HBDSP antibodies, and anti-GAPDH antibodies served as the loading control. All assays were performed in triplicate. (**B**) Apoptosis assay. HepG2.2.15 and HBV-infected HepG2-NTCP cell lines were, respectively, transfected with the phouge-HBDSP or phouge for 48 h followed by flow cytometry analysis using PE Annexin-V staining or FITC Annexin-V staining. The ratios of apoptosis in HBDSP-transfected HepG2.2.15 and HepG2-NTCP cells were, respectively, upregulated by 127.68% and 48.03%, compared with the empty vector control. The Apoptosis Inducers Kit was used as a positive control. All assays were performed in triplicate. Data are presented as the means ± SD. *P < 0.05 compared to the control. (**C**) Western blot analysis of the *p53*-mediated apoptotic signaling pathway. HepG2.2.15 cells and HBV-infected HepG2-NTCP cells were transfected with phouge-HBDSP or phouge for 48 h, and the levels of p53, p-p53 (Ser15), Bax, cleaved-caspase-3, Bcl-2, and HBDSP protein were detected by Western blot analysis. Relative protein quantitative values were normalized to the corresponding internal control tubulin. The relative levels of proteins expression were obtained by a comparison with the empty phouge plasmids, which was set to 1. All assays were performed in triplicate. Data are shown as the means ± SD. *P < 0.05 compared to the control. (**D**) HBV DNA quantification. HepG2.2.15 cells and HBV-infected HepG2-NTCP cells were treated with 20 µM PFTα or DMSO for 6 h following transfection with phouge-HBDSP or phouge for 42 h, and the number of HBV DNA copies in the culture supernatants was quantified. All assays were performed in triplicate. Data are shown as the means ± SD. *P < 0.05 compared to the control. (**E and F**) HBsAg and HBeAg detection. HepG2.2.15 cells and HBV-infected HepG2-NTCP cells were treated with 20 µM PFTα or DMSO for 6 h following transfection with phouge-HBDSP or phouge for 42 h, and the relative HBsAg levels (**E**) and HBeAg levels (**F**) in the culture supernatants were quantified. The phouge-transfected and DMSO-treated groups were set as 1 for comparison. All assays were performed in triplicate. Data are shown as the means ± SD. *P < 0.05 compared to the control.

To measure the expression levels of HBDSP in HBV-replicating and HBV-infected cells, antibodies specific to HBDSP were designed and synthesized against the first spliced donor-acceptor sites of HBDSP. As shown in Fig. 7A, the expression levels of HBDSP in HBV-replicating HepG2.2.15 and HepAD38 cells, as well as in HBV-infected HepG2-NTCP cells could be detected using anti-HBDSP antibodies by Western blot assays. To further determine whether HBDSP has the pro-apoptotic effects in HBV-replicating cells, HBDSP was overexpressed in HepG2.2.15 cells. Additionally, given that HepAD38 cells, like HepG2.2.15 cells, are both replication models, we have specifically included the infection cell model to assess the overexpression of HBDSP in HBV-infected HepG2-NTCP cells. The results in Fig. 7B and 7C showed that HBDSP overexpression promoted cellular apoptosis and increased the expression levels of p53, p-p53 (Ser15), p53 signaling pathway-related Bax, and cleaved-caspase-3 but decreased the expression levels of Bcl-2 in both HBV-replicating HepG2.2.15 cells and HBV-infected HepG2-NTCP cells. Furthermore, the levels of secreted HBV DNA, HBsAg, and HBeAg in culture supernatants were, respectively, quantified. The results revealed that HBDSP promotes the secretion of HBV DNA (Fig. 7) and increases the expression of HBsAg (Fig. 7E) and HBeAg (Fig. 7F), while the levels of HBV DNA, HBsAg, and HBeAg were decreased after treating with p53 inhibitor PTFα. Taken together, these results indicate that HBDSP can activate *p53*-mediated apoptotic signaling pathway in HBV-replicating and HBV-infected cells and increase the production of HBV progeny.

## DISCUSSION

Chronic HBV infection represents a global public health threat that increases the risk of developing CHB, liver cirrhosis, liver failure, and HCC (29, 30). Transcription of the 3.2-kb partially double-stranded HBV DNA genome generates a 3.5-kb pre-core/pgRNA and three sub-genomic RNAs with a length of 2.4-, 2.1-, and 0.7-kb, respectively (31). The HBV genome replicates through the reverse transcription of the 3.5-kb pgRNA (32). Besides 3.2-kb of full-length HBV genome, it has been reported that defective HBV spliced genomes were generated from the reverse transcription of HBV pgRNA in the serums of CHB patients and were associated with liver diseases (33, 34). The most frequently detected spliced HBV genomes in the serums of patients with CHB are 2.2 kb singly and doubly spliced defective HBV genomes. In previous studies, it has been demonstrated that the 2.2-kb singly spliced variant and its expressed protein HBSP could induce Huh7 and HepG2 cell apoptosis and further exacerbate the development of hepatitis (6, 35). It has also been shown that HBSP is involved in viral replication, severity of liver fibrosis (9), and can cause liver damage by inducing specific CTL activity (10). However, some studies have shown contradictory results, indicating that the 2.2-kb singly spliced variants could efficiently inhibit HBV replication (36), and the role of T-cell response to HBSP in liver damage of chronic HBV patients was relatively weak (11). In addition, HBSP could inhibit Fas-mediated hepatocyte apoptosis via PI3K/Akt (37) as well as promote the invasion and migration of hepatocytes (38). Few studies have focused on the potential pathogenicity of 2.2-kb HBV doubly spliced defective genome and its encoded HBDSP protein in the progression of CHB. We reveal a novel mechanism here by which HBDSP can induce hepatoma cell apoptosis via *p53* transcriptional transactivation.

Hepatocyte apoptosis is considered to be a prominent pathological feature of chronic liver diseases (15). HBV infection results in numerous changes in hepatocytes, which could directly or indirectly affect various cellular processes, including apoptosis (39, 40). The mechanisms underlying virus-induced apoptosis are complex and multifaceted. HBV-infected hepatocyte apoptosis may have dual effects. It may contribute to antiviral defense mechanisms by limiting viral replication and spreading through the elimination of infected cells (41), but it may also support the release and spread of HBV particles to neighboring cells, associating with fibrogenesis and chronic liver dysfunction (42, 43). The HBx might maximize the production of viral progeny during early stages of hepatocyte infection and subsequently induce apoptosis to facilitate the efficient release of HBV particles and reduce the antiviral inflammatory responses (44 – 47). HBSP could induce the apoptosis of hepatoma cells, which might lead to viral particles diffusion and diminish the immune neutralization, further exacerbating the development of hepatitis (6, 35, 48). Other truncated HBV variants, including large surface proteins and pre-core mutants, have been demonstrated to be implicated in the HCC development process by supporting virion secretion or increasing viral replication (49, 50).

Our results revealed that HBDSP encoded by the 2.2-kb doubly spliced HBV variants exhibited pro-apoptotic effects in the wt-*p53* HepG2 and SMMC-7721 cell lines but did not exhibit any effects in another two mut-*p53* HCC cell lines (Huh7 and MHCC-97H cells). HepG2 and SMMC-7721 cells were treated with the p53 inhibitor, PFTα, and p53 siRNA to validate the effects of wt-*p53* in HBDSP-induced apoptosis. It has been well established that the *p53* gene is a crucial regulator of the apoptosis pathway (16) and is also the most frequently somatically mutated gene in over 50% of human cancers, albeit the frequency of the *p53* mutation remains low in HCC (51). Mut-*p53* commonly loses the activities and functions of wt-*p53*, thereby failing to induce apoptosis (26). This finding is in-line with our finding that HBDSP had no pro-apoptotic effect in mut-*p53* Huh7 and MHCC-97H cell lines. It has been reported that wt-*p53* can mediate apoptosis via a linear pathway involving Bax and caspase-3 activation, thereby limiting cellular proliferation (52). Furthermore, phosphorylation of p53 at Ser15 has been demonstrated as one of the most crucial phosphorylation sites, which could activate p53 and trigger a cascade of additional p53 phosphorylation events, including Ser46 phosphorylation, thereby further contributing to the induction of apoptosis (53, 54). We also demonstrated that HBDSP upregulated the levels of p53 and p-p53 (Ser15) expression and activated the *p53-*mediated apoptotic signaling pathway by upregulating the levels of Bax and cleaved-caspase-3 expression and downregulating the levels of Bcl-2 expression, which could be reversed by using p53 inhibitor PFTα or p53 siRNA.

It has been documented that HBx, a viral transactivator with oncogenic potentials, could induce cellular apoptosis in both a *p53-*dependent (23, 55) and *p53-*independent (46, 47) manner. Our previous study also revealed that HBDSP is a pleiotropic transactivator, which exerts transactivational abilities through AP-1- or C/EBP-binding sites, and the 48–75 amino acid residues within HBDSP were found to be crucial for its transactivational activities (12). In the present study, we identified the critical −595 nt/−360 nt regions in the *p53* promoter, with which ETS1, GATA2, and YY1 could bind and facilitate *p53* transcriptional activation via HBDSP. Previous studies have shown that ETS1 can regulate p53 expression through the ETS-binding site palindrome in the −391 nt to −379 nt regions of the *p53* promoter (56). GATA2 has been reported to bind to the *p53* promoter regions from −240 nt to −146 nt (57). Moreover, YY1 has been identified to interact with the *p53* promoter in the −102 nt to −96 nt regions (58). It is important to mention that the present results showed that the binding regions of ETS1, GATA2, and YY1 with the *p53* promoter induced by HBDSP, as verified by EMSA and ChIP analysis, were inconsistent with abovementioned literature.

Additionally, we demonstrated detailed mechanisms by which HBDSP can indirectly regulate the *p53* promoter by ETS1, GATA2, and YY1. Our results indicate that HBDSP has no transcriptional activation effects on the levels of ETS1, GATA2, and YY1, and HBDSP cannot directly bind with ETS1, GATA2, and YY1. However, HBDSP could promote the nuclear translocation of ETS1, GATA2, and YY1. The detailed mechanisms by which HBDSP leads to the aggregation of ETS1, GATA2, and YY1 in the nucleus require further study.

In the present study, HBDSP could be successfully detected in both HBV-replicating HepG2.2.15 and HepAD38 cells, as well as HBV-infected HepG2-NTCP cells. Considering that HBSP does not have the above-referenced antibody epitope against HBDSP and Western blot assays had been done to clarify that the antibodies against HBDSP could not bind with HBSP (data not shown), our findings suggest that specific antibodies targeting HBDSP may be utilized for quantitatively measuring the levels of HBDSP protein in the sera of HBV-infected patients. This is of significant importance for distinguishing different stages of hepatitis B.

Furthermore, we also demonstrated that HBDSP is capable of promoting cellular apoptosis and activating the *p53*-mediated apoptotic signaling pathway in both HBV-expressing HepG2.2.15 cells and HBV-infected HepG2-NTCP cells. And HBDSP increases the secretion of HBV DNA, HBsAg, and HBeAg into the culture supernatants of HepG2.2.15 cells and HBV-infected HepG2-NTCP cells, which could be reversed by using p53 inhibitor PFTα. In CHB patients, HBV DNA primarily derive from mature infectious particles and reflect viral replication (59). And the production of a large amount of viral proteins, such as HBsAg and HBeAg, is typically associated with HBV persistence (60). In addition, HBsAg levels in serums serve as complementary indicator of viral load, which not only reflect the transcription of cccDNA or the translation of mRNA but also reflect host immune control over HBV infection (59). The increased HBV DNA, HBsAg, and HBeAg indicate the persistence of HBV in hepatocytes, triggering activation of the immune system, leading to inflammatory response, and causing liver damage (59 – 61). Therefore, it was speculated that HBDSP may be involved in the occurrence and development of HBV-related CHB. Notably, the relative differences observed in both the luciferase assays used to validate the transactivational effects of HBDSP and the qPCR and enzyme-linked immunosorbent assay (ELISA) assays employed to evaluate HBV progeny seem a little small. Actually, in our previous study (12), the transactivational function of HBDSP was qualitatively detected through a yeast two-hybrid assay. Besides, quantitatively assessing using reporter gene assays also revealed that the transactivational effects of HBDSP were consistently relatively weak. This may be attributed to the fact that the hepatocarcinogenesis consequences triggered by HBDSP are subtle and gradual in nature.

In summary, our data demonstrate that HBDSP induces wt-*p53-*mediated apoptosis in HCC through promoting the nuclear translocation of ETS1, GATA2, and YY1 (as shown in Fig. 6D). A number of viruses, including human papillomavirus, human immunodeficiency virus, and human T-cell leukemia virus type 1 and HBV, are known to have developed pro-apoptotic strategies facilitating viral propagation and promoting infection by permitting efficient viral particles release from host cells (41, 62 – 64). Based on our findings, we speculate that HBV particles released by HBDSP-induced apoptotic cells may be a key factor in the persistence of HBV in CHB. Therefore, the present study may provide a potential therapeutic option for preventing the further deterioration of HBV-infected livers by blocking HBDSP-induced apoptosis, thereby reducing HBV virulence.

## MATERIALS AND METHODS

### Plasmids and cloning

The p53 gene (NM_000546.6) sequences were obtained from the NCBI database (https://www.ncbi.nlm.nih.gov), and the *p53* promoter sequences were determined using the UCSC Genome Browser (https://genome.ucsc.edu) tool. The *p53* promoter construct pGL4.10-p53-2000 was generated by General Biol (Anhui) Co., Ltd. A range of *p53* promoter constructs with 5′-deletion, including pGL4.10-p53-1509, pGL4.10-p53-1005, pGL4.10-p53-497, pGL4.10-p53-814, pGL4.10-p53-595, pGL4.10-p53-360, and pGL4.10-p53-195, were constructed using pGL4.10-p53-2000 as the template. The corresponding fragments generated by PCR were cloned into the *Kpn* I and *Xho* I (New England BioLabs, USA) sites of the pGL4.10 vector (Promega, USA). pGL4.10-p53-595 was used to construct ETS1-, GATA2-, and YY1-binding site-mutated constructs, including pGL4.10-p53-595-ETS1-mut, pGL4.10-p53-595-GATA2-mut1, pGL4.10-p53-595-GATA2-mut2, pGL4.10-p53-595-GATA2-mut3, pGL4.10-p53-595-YY1-mut1, and pGL4.10-p53-595-YY1-mut2. The wild-type and mutated nucleotides for the putative ETS1, GATA2, and YY1 binding sites are listed in Table S1.

Recombinant plasmids expressing HBDSP genes (GenBank No. FJ151414.1), phouge-HBDSP, were generated by ligation of HBDSP into the *Xba* I and *Bam*H I (New England BioLabs) sites of the pCDH-EF1-MCS-T2A-Puro vector (System Biosciences, USA, abbreviated as phouge in this manuscript), and pDsRed-HBDSP were obtained by ligation of HBDSP into the *Xho* I and *Sal* I (New England BioLabs) sites of pDsRed-monomer-hyg-N1 vector (Clontech, USA, abbreviated as pDsRed). The plasmids phouge-HBDSPΔ48–75 with 48–75 amino acid deletions of HBDSP were constructed by inserting into the *Xba* I and *Bam*H I sites of the phouge vector. To construct plasmids encoding the transcriptional factors, including pcDNA3.1-ETS1 (NM_001143820.2), pcDNA3.1-GATA2 (NM_032638.5), and pcDNA3.1-YY1 (NM_003403.5), PCR-generated products were inserted into the *Nhe* I and *Kpn* I (New England BioLabs) sites of the pcDNA3.1/Hygro(+) vector (Invitrogen, USA). The ETS1-, GATA2-, and YY1-expressing plasmids fused with GFP (pAcGFP-ETS1, pAcGFP-GATA2, and pAcGFP-YY1) were constructed by inserting the PCR-generated fragments into the *Nhe* I and *Kpn* I (New England BioLabs) sites of the pAcGFP1-Hyg-N1 plasmid (Clontech). Table S2 lists primer sequences for PCR amplification.

### Cell cultures and transfection

The human hepatoblastoma cell line, HepG2 (American Type Culture Collection, USA), was maintained in Minimal Eagle’s Medium (Thermo Scientific, USA) containing 10% (vol/vol) fetal bovine serum (FBS; PAN, Germany). Human hepatocellular carcinoma cell lines (SMMC-7721, Huh7, and MHCC-97H) were obtained from the Cell Bank of Type Culture Collection (Chinese Academy of Sciences, Shanghai, China) and were grown in Dulbecco’s Modified Eagle’s Medium (DMEM; Thermo Scientific) with 10% FBS. HepAD38 cells were maintained in DMEM with 10% FBS and 6 µg/mL tetracycline (Tet) (MCE, USA). When Tet was removed from the culture medium, the HepAD38 cells secreted HBV virions. HepG2-NTCP cells were maintained in DMEM with 10% FBS. HepG2.2.15, stably transfected with HBV, were maintained in DMEM containing 10% FBS and 400 µg/mL G418 (Sigma-Aldrich, USA). All cells were grown at 37°C in a humidified atmosphere containing 5% CO_2_. The Lipofectamine 3000 (Invitrogen) reagents were used to transfect plasmids according to the supplier’s recommendations.

### HBV production and infection

HBV particles were harvested from the culture supernatants of HepAD38 cells, and then HepG2-NTCP cells were infected according to the previously established process (65, 66). Briefly, supernatants were carefully gathered and mixed with 8% polyethylene glycol (PEG) 8000 (Sigma-Aldrich), and this mixture were then rotated overnight at 4°C for 12–16 h, following by centrifuging at 4°C, 10,000 × *g* for 30 min. The precipitate containing HBV particles was subsequently redissolved in DMEM at 1% relative to the original sample volume. For the infection process, HepG2-NTCP cells were seeded into 35 mm collagen-I-coated plates in DMEM containing 10% FBS and 1 mg/mL doxycycline (TaKaRa, Japan) for 72 h. Then, the cells were infected with HBV particles at the 200 multiplicities of infection (MOI) in the presence of 8% PEG 8000 supplement with 1.5% DMSO (Sigma-Aldrich).

### Adenovirus infection

HBDSP-overexpressing adenoviruses (Ad-HBDSP) and an empty control (Ad-GFP) were constructed as described previously (67). HepG2, SMMC-7721, Huh7, and MHCC-97H cells were seeded into six-well culture plates at a corresponding density and infected with Ad-HBDSP or Ad-GFP recombinant adenoviruses at an MOI of 20, 50, 60, or 100 or the indicated doses, respectively.

### RNA interference

The siRNA duplexes for p53, ETS1, GATA2, YY1, and control were synthetized by GenePharma Company (China). Table S3 lists the siRNA sequences. The Lipofectamine 3000 reagents were used to transfect the siRNA duplexes into cells following the manufacturer’s recommendations.

### Drugs and treatment

HepG2 and SMMC-7721 cell lines were treated with 20 µM PFTα (Selleck, USA) and dissolved in DMSO for 6 h after infection with Ad-HBDSP for 66 h to inhibit p53 expression. HepG2.2.15 cells and HBV-infected HepG2-NTCP cells were treated with 20 µM PFTα for 6 h after transfection with phouge-HBDSP for 42 h to inhibit p53 expression.

### Apoptosis assay

A PE Annexin-V Apoptosis Detection Kit and an FITC Annexin-V Apoptosis Detection Kit (BD Pharmingen, USA) were used to quantify apoptotic cells as described by the manufacturer’s instructions. And cells treated with an Apoptosis Inducers Kit (Beyotime, China) were used as positive controls for apoptosis assays according to the previously described procedure (68). Briefly, HepG2, SMMC-7721, Huh7, MHCC-97H, HepG2.2.15, or HBV-infected HepG2-NTCP cells were trypsinized and collected by centrifugation at 800 × *g* for 5 min. The cells were resuspended in 1× binding buffer after washing with 1× PBS. Apoptosis rates were evaluated using a FACSVerse flow cytometer (BD Biosciences, USA) after adding PE Annexin V or FITC Annexin V and 7-AAD to the buffer for 20 min in the dark. The data were analyzed with FACSuite software (BD Biosciences).

### Western blot analysis

The cell lysis buffer (Beyotime) containing 1× phenylmethanesulfonyl fluoride (PMSF; Beyotime) and 1× cocktail protease inhibitors (MCE, USA) was used to lyse cells for 30 min on ice to obtain the total cellular proteins. The NE-PER Nuclear and Cytoplasmic Extraction Reagents (Thermo Scientific) were used to obtain cytoplasmic and nuclear proteins according to the manufacturer’s recommendations. A BCA Protein Assay Kit (Pierce Biotechnology, Appleton, USA) was used to quantify proteins. The 12% SDS-PAGE gels were used to separate an equal amount of protein, and the polyvinylidene difluoride membranes (Beyotime) were used to transfer proteins. Then, the membranes were blocked with 1% BSA for 2 h and incubated with specific primary antibodies overnight at 4℃. The antibodies included anti-p53 (abcam, UK), anti-p-p53 (Ser15) (Proteintech, China), anti-Bax (CST, USA), anti-cleaved-caspase-3 (CST), anti-Bcl-2 (CST), anti-Flag (Sigma), anti-Tubulin (CST), anti-ETS1 (CST), anti-GATA2 (Abcam), anti-YY1 (CST), anti-GAPDH (CST), and anti-histone H3 (Proteintech). The monoclonal anti-HBDSP specific antibodies were designed to recognize HBDSP by targeting the first spliced donor-acceptor sites (i.e., around the 2,447 nt to 2,935 nt spliced sites) of 2.2-kb doubly spliced variants of HBV. The membranes were washed with 1× TBST for three times and incubated with HRP-conjugated secondary antibodies for 1 h. An enhanced chemiluminescence (ECL) substrate solution (Amersham, UK) was added to the membranes, and the levels of protein expression were measured using an Image Quant LAS 4000 mini system (GE Healthcare, Waukesha, WI, USA). The changes in the levels of protein expression were analyzed with ImageJ software (Rawak Software, Inc. Germany).

### qRT-PCR

The TRIzol Reagent (Invitrogen) was used to isolate the total RNA as described by the manufacturer’s instructions. Next, the total RNA and an Evo M-MLV RT Kit (Accurate Biology, China) were used to synthesize cDNA. A qRT-PCR assay was performed to determine the transcription levels of *p53* by using an SYBR Green Pro Taq HS qPCR Kit (Accurate Biology) following the manufacturer’s recommendations. GAPDH primers were used as a reference to normalize the data, and the 2^−ΔΔCt^ method was used to calculate the relative expression levels of *p53* mRNA as previously described (69). Table S2 lists the specific primer sequences for qRT-PCR.

### Luciferase reporter assay

The Bright-Glo Luciferase Assay System (Promega) was used to perform luciferase assays following the supplier’s recommendations. The post-transfected cells were washed three times with 1× PBS and lysed in 1× Glo Lysis Buffer (Promega) for 15 min. Next, the lysates were harvested by centrifugation, and BCA Protein Assay Kit was used to quantify the supernatants. Equal amounts of supernatants (30 µg) were taken from each sample and supplemented with 1× Glo Lysis Buffer to an equal volume (100 µL), which were used to test the luciferase activities by using an Orion II Microplate Luminometer (Berthold Detection Systems, German).

### EMSA

The NE-PER Nuclear and Cytoplasmic Extraction Reagents (Thermo Scientific) were used to isolate nuclear extracts from HepG2 cells or HBDSP-transfected HepG2 cells following the supplier’s instructions. Next, an EMSA was performed using the LightShift Chemiluminescent EMSA Kit (Thermo Scientific) following the supplier’s recommendations. Biotin-labeled double-stranded oligonucleotides containing the consensus sequences of ETS1-, GATA2-, or YY1-binding sites were used as probes. Unlabeled double-stranded oligonucleotides were used as competitor probes (cold probes), and site-directed mutated oligonucleotides were used as a negative control (cold mutated probes). All single-stranded oligonucleotides were synthesized by SunYa Biotechnology (Fuzhou) Co., Ltd. Table S4 lists the oligonucleotide sequences of probes. After heating to 95°C for 2 min and then cooling naturally to room temperature, an equal amount of the complementary single-stranded oligonucleotides was annealed to produce double-stranded oligonucleotides. Incubations were performed with biotin-labeled probes and nuclear extracts in poly (dI-dC) and 1× binding buffer. Prior to adding of the biotin-labeled probes, an excess of cold or cold-mutated probes was pre-incubated with the reaction mixtures for a competition assay. A super-shift assay was performed with anti-ETS1, -GATA2, or -YY1 antibodies to confirm the specificity of the nuclear proteins combined with specific recognition sites. Following the incubation, 6.0% non-denatured PAGE gels were used to separate DNA-protein complexes, and the bands were transferred to nylon membranes (Beyotime). The membrane was blocked with 1× blocking buffer after cross-linking by UV irradiation and incubated with streptavidin-HRP for 15 min. Then, the membrane was washed with 1× washing buffer four times. The membrane was incubated following the addition of 1× detection buffer and working substrate solution, respectively. Finally, the membrane was imaged and photographed with an Image Quant LAS 4000 mini system.

### ChIP assay

A SimpleChIP Enzymatic Chromatin IP Kit (Magnetic Beads) (CST) was used to perform ChIP assays as the supplier’s instructions described. Briefly, the cells were cross-linked with 1% formaldehyde and terminated by a 1× glycine solution. The cells were scraped with cold 1× PBS containing a 1× cocktail of protease inhibitors and harvested by centrifugation. The cells were lysed with the mixture of 1× buffer A, 1× buffer B, and 1× ChIP buffer. The extracted chromatin was digested and fragmented to 150 bp to 900 bp using micrococcal nuclease followed by sonication. DNA-protein complexes of obtained chromatin fragments were subjected to immunoprecipitation with specific anti-ETS1, -GATA2, -YY1, or -Flag antibodies or with negative control anti-IgG antibodies or positive control anti-histone H3 antibodies. Protein G magnetic beads were added followed by an incubation at 4℃ for 2 h. Finally, 1× ChIP elution buffer was added to obtain the immunocomplexes. Following reverse cross-linking with proteinase K and NaCl and DNA purification, immunoprecipitated DNA was used as a template for PCR or qPCR to amplify the fragments containing the binding site of ETS1, GATA2, or YY1 in the *p53* promoter. Table S2 lists the specific primer sequences for PCR or qPCR amplification.

### Co-immunoprecipitation

Post-transfection with phouge-HBDSP for 48 h, the lysis buffer was used to lyse cells on ice. Next, the lysates were centrifuged, and the collected supernatants were quantified. Supernatants were subjected to Protein A/G Agarose (SantaCruz, USA) beads and anti-Flag antibodies or anti-IgG at 4℃ overnight. The agarose beads were washed with lysis buffer three times followed by boiling and centrifugation. The supernatants were separated on 10% SDS-PAGE gels and detected by Western blot assays using specific anti-ETS1, -GATA2, -YY1, -TFII-I (CST), -Flag, or -GAPDH antibodies. The positive control indicator TFII-I (general transcription factor II, I), a transcription factor, has been verified to interact with HBDSP both *in vitro* and *in vivo* in our previous study (67).

### Confocal microscopy

After co-transfection with pDsRed-HBDSP and pAcGFP-ETS1, -GATA2, or -YY1 for 48 h, the cells were washed with cold 1× PBS three times and fixed with 4% paraformaldehyde. After the cells were washed three times with cold 1× PBS, Hoechst 33342 (Beyotime) was added to stain the cells for 5–10 min. Next, the 1× PBS was used to wash cells five times. Finally, the cells were examined and photographed with a Leica TCS SP8 X confocal system (Leica, Germany).

### HBV DNA quantification

The Hepatitis B Viral DNA Quantitative Fluorescence Diagnostic Kit (Sansure Biotech, China) was used to quantify the secreted HBV DNA in the culture supernatants of HepG2.2.15 cells and HBV-infected HepG2-NTCP cells following the supplier’s recommendations. Briefly, the culture supernatants of HepG2.2.15 cells and HBV-infected HepG2-NTCP cells were collected after transfection with phouge-HBDSP for 42 h and then treatment with 20 µM PFTα for 6 h. Subsequently, the supernatants were centrifuged at 800 × *g* for 5 min, and the resulting pellets were discarded. The extracellular HBV DNA copies in the supernatants were then quantified by qPCR using the Agilent Technologies Stratagene Mx3000P (Agilent Technologies, USA).

### ELISA detection

To detect the secreted HBsAg and HBeAg levels in the culture supernatants of HepG2.2.15 cells and HBV-infected HepG2-NTCP cells, the human HBsAg and HBeAg ELISA Kits (Andy Gene, China) were utilized according to the supplier’s instructions. Briefly, after transfection with phouge-HBDSP for 42 h and treatment with 20 µM PFTα for 6 h, the culture supernatants of HepG2.2.15 cells and HBV-infected HepG2-NTCP cells were collected. Subsequently, the extracellular HBsAg and HBeAg in the supernatants were, respectively, quantified using the EnSight Multi-Mode Microplate Reader (PerkinElmer, USA).

### Statistical analysis

SPSS 22.0 software (SPSS Inc, USA) was used to perform statistical analysis. All the quantitative data are presented as the mean ± SD of experiments with three independent repeats. Student’s *t*-test was used to compare differences between two groups. *P* < 0.05 was considered to be statistically significant.

## SUPPLEMENTAL MATERIAL

Fig.S1 to S5, Tables S1 to S4 jvi.01087-23-s0001.pdf Supplemental material.

ASM does not own the copyrights to Supplemental Material that may be linked to, or accessed through, an article. The authors have granted ASM a non-exclusive, world-wide license to publish the Supplemental Material files. Please contact the corresponding author directly for reuse.
