# Supplementary material for: Hepatitis B doubly spliced protein (HBDSP) promotes hepatocellular carcinoma cell apoptosis via ETS1/GATA2/YY1-mediated p53 transcription
Source: J Virol. 2023 Nov 6;97(11):e01087-23. doi: 10.1128/jvi.01087-23 (PMC10688342; doi:10.1128/jvi.01087-23)
Supplement: Fig.S1 to S5, Tables S1 to S4 — Supplemental material. [file jvi.01087-23-s0001.pdf]

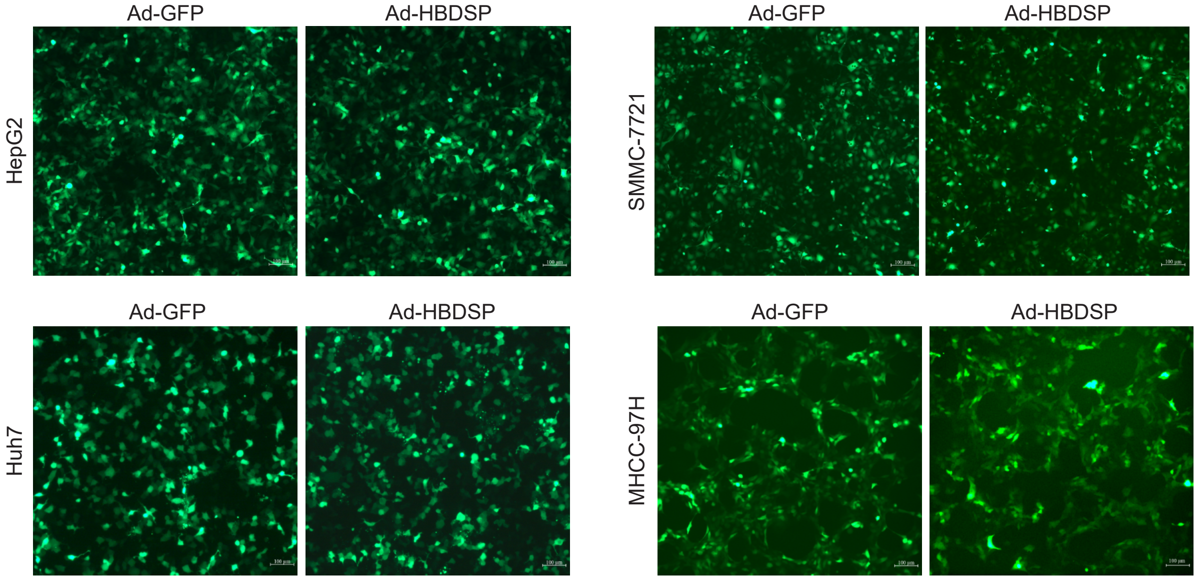
Ad-GFP
Ad-HBDSP
HepG2
Ad-GFP
Ad-HBDSP
SMMC-7721
Ad-GFP
Ad-HBDSP
Huh7
Ad-GFP
Ad-HBDSP
MHCC-97H

Fig S1

FIGURE S1. Adenovirus infection. (A and B) Fluorescence microscopy image. HepG2, SMMC-7721, Huh7, and MHCC-97H cell lines were respectively infected with Ad-HBDSP or Ad-GFP at MOIs of 20, 50, 60, or 100 for 72 h followed by a photograph using a Fluorescence Microscope.

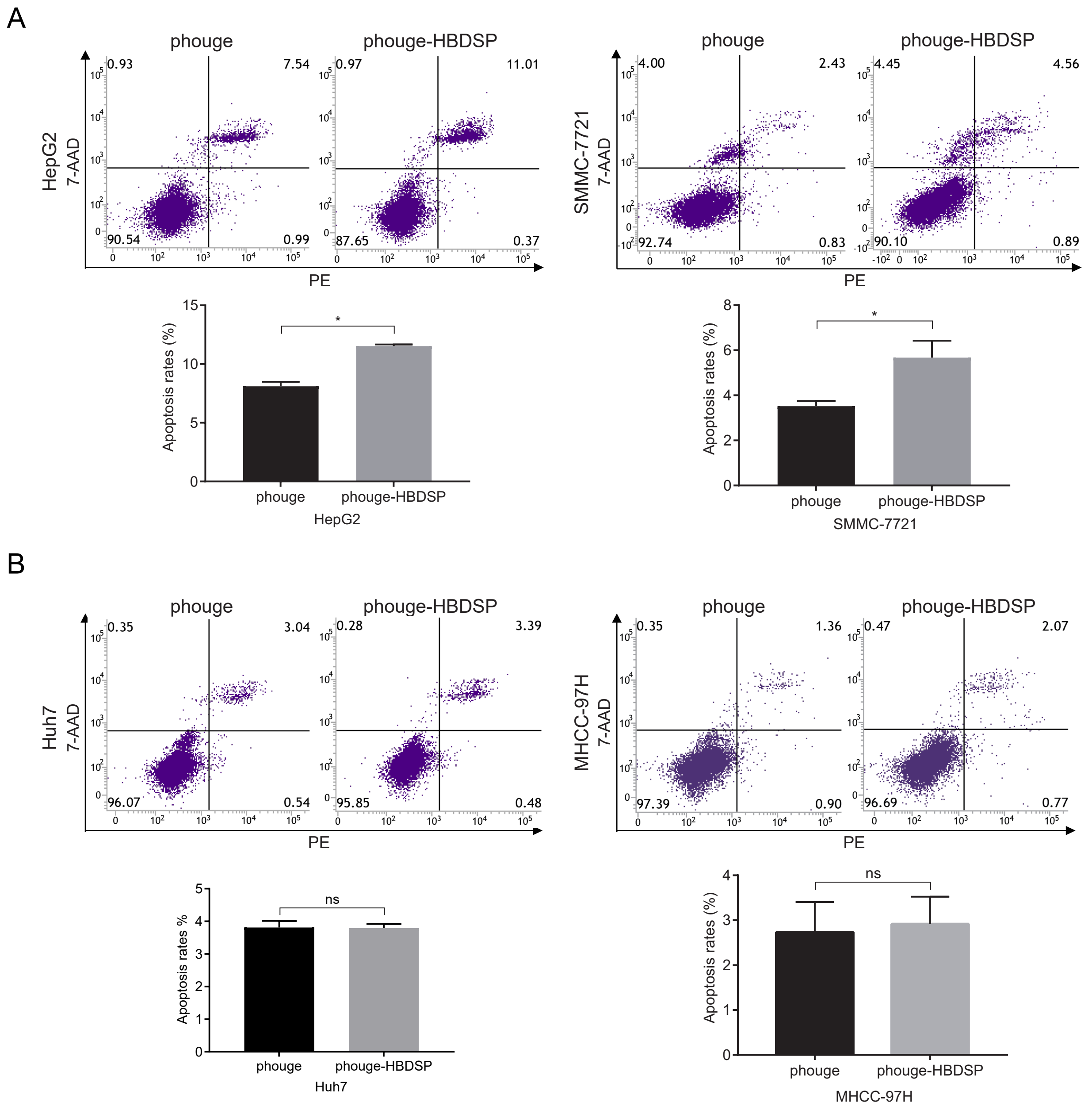
A
phouge
phouge-HBDSP
HepG2
7-AAD
PE
0.93
7.54
90.54
0.99
0.97
11.01
87.65
0.37
SMMC-7721
4.00
2.43
92.74
0.83
4.45
4.56
90.10
0.89
Apoptosis rates (%)
*
HepG2
SMMC-7721
B
Huh7
0.35
3.04
96.07
0.54
0.28
3.39
95.85
0.48
MHCC-97H
0.35
1.36
97.39
0.90
0.47
2.07
96.69
0.77
Apoptosis rates %
ns
Huh7
MHCC-97H

Fig S2

FIGURE S2. The effects of transiently transfected-HBDSP on hepatocellular apoptosis. (A and B) Apoptosis assay with plasmids transfection. HepG2, SMMC-7721, Huh7, and MHCC-97H cell lines were respectively transfected with phouge-HBDSP or phouge for 48 h followed by an analysis using a PE Annexin-V staining and flow cytometry. The ratio of apoptosis in HBDSP-transfected HepG2 and SMMC-7721 cells were respectively up-regulated by 49.68% and 40.78%, compared with the control, whereas the Huh7 and MHCC-97H cell lines displayed no significant changes. All assays were performed in triplicate. Data are presented as the means ± SD. $*P < 0.05$ compared to the control. ns, non-significant.

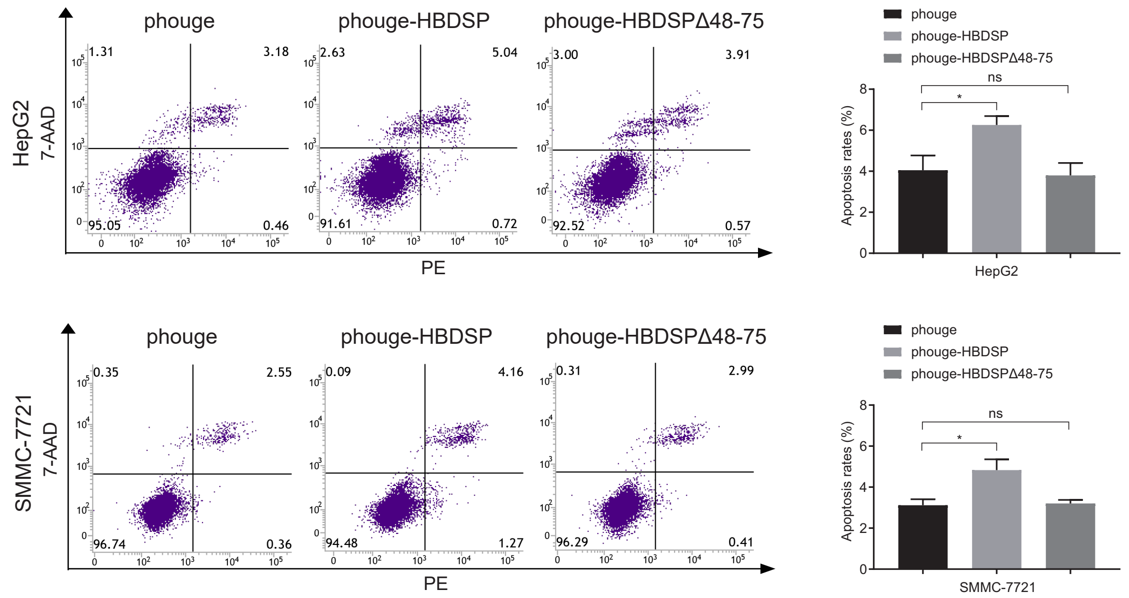

phouge
phouge-HBDSP
phouge-HBDSPΔ48-75
HepG2
7-AAD
PE
1.31
3.18
95.05
0.46
2.63
5.04
91.61
0.72
3.00
3.91
92.52
0.57
Apoptosis rates (%)
ns
*
HepG2
SMMC-7721
0.35
2.55
96.74
0.36
0.09
4.16
94.48
1.27
0.31
2.99
96.29
0.41

Fig S3

FIGURE S3. HBDSP induced hepatocellular apoptosis via the transactivation domain of its 48-75 amino acids residues. (A and B) Apoptosis assay. HepG2 and SMMC-7721 cell lines were respectively transfected with phouge-HBDSP, phouge-HBDSPΔ48-75 or phouge for 48 h followed by an analysis using a PE Annexin-V staining and flow cytometry. The ratios of apoptosis in HBDSP-transfected HepG2 and SMMC-7721 cells were up-regulated by 54.49% and 55.08% respectively, compared to the empty control, whereas there were no significant changes in the apoptotic rates of the HBDSPΔ48-75 mutant group compared to the empty control. All assays were performed in triplicate. Data are presented as the means ± SD. $^*P < 0.05$ compared to the control. ns, non-significant.

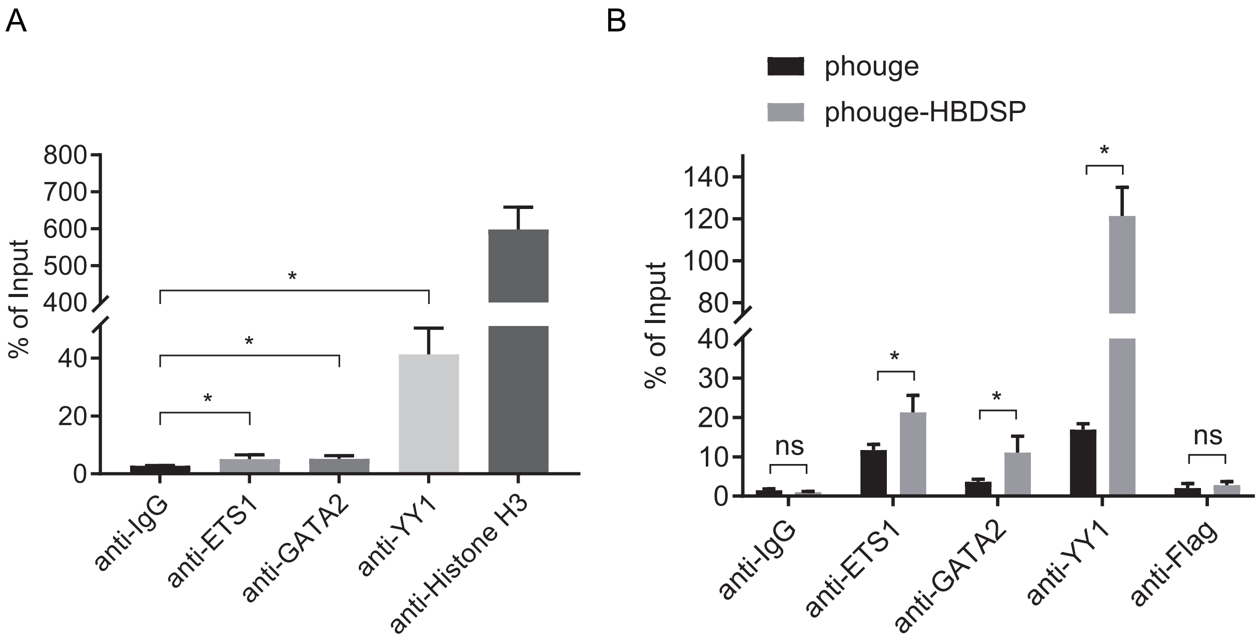
A
800
700
600
500
400
40
20
0
% of Input
*
*
*
anti-IgG
anti-ETS1
anti-GATA2
anti-YY1
anti-Histone H3
B
phouge
phouge-HBDSP
140
120
100
80
40
30
20
10
0
% of Input
ns
*
*
*
ns
anti-IgG
anti-ETS1
anti-GATA2
anti-YY1
anti-Flag

Fig S4

FIGURE S4. HBDSP enhanced the enrichment of transcription factors ETS1, GATA2, and YY1 to binding with p53 promoter *in vivo.* (A) ChIP assay of ETS1, GATA2, and YY1 binding to the p53 promoter in HepG2 cells. Chromatin from HepG2 cells were immunoprecipitated with the anti-ETS1, -GATA2, or -YY1 antibodies. The total extracted DNA (2% Input) prior to immunoprecipitation, as well as the immunoprecipitated and purified DNA, were amplified by qPCR using specific primers targeting the -595nt/-360nt region of the p53 promoter. Pre-blocked protein A/G or normal rabbit IgG or Histone H3 antibodies were used as controls. (B) ChIP assay of ETS1, GATA2, and YY1 binding to the p53 promoter in HBDSP-transfected HepG2 cells. Chromatin from HepG2 cells transfected with or without phouge-HBDSP were immunoprecipitated with the anti-ETS1, -GATA2, -YY1, or -Flag antibodies. The total extracted DNA (2% Input) prior to immunoprecipitation, as well as the immunoprecipitated and purified DNA, were amplified by qPCR. Normal rabbit IgG antibodies were used as negative controls. All assays were performed in triplicate. Data are presented as the means ± SD. $^{*}P < 0.05$ compared to the control. ns, non-significant.

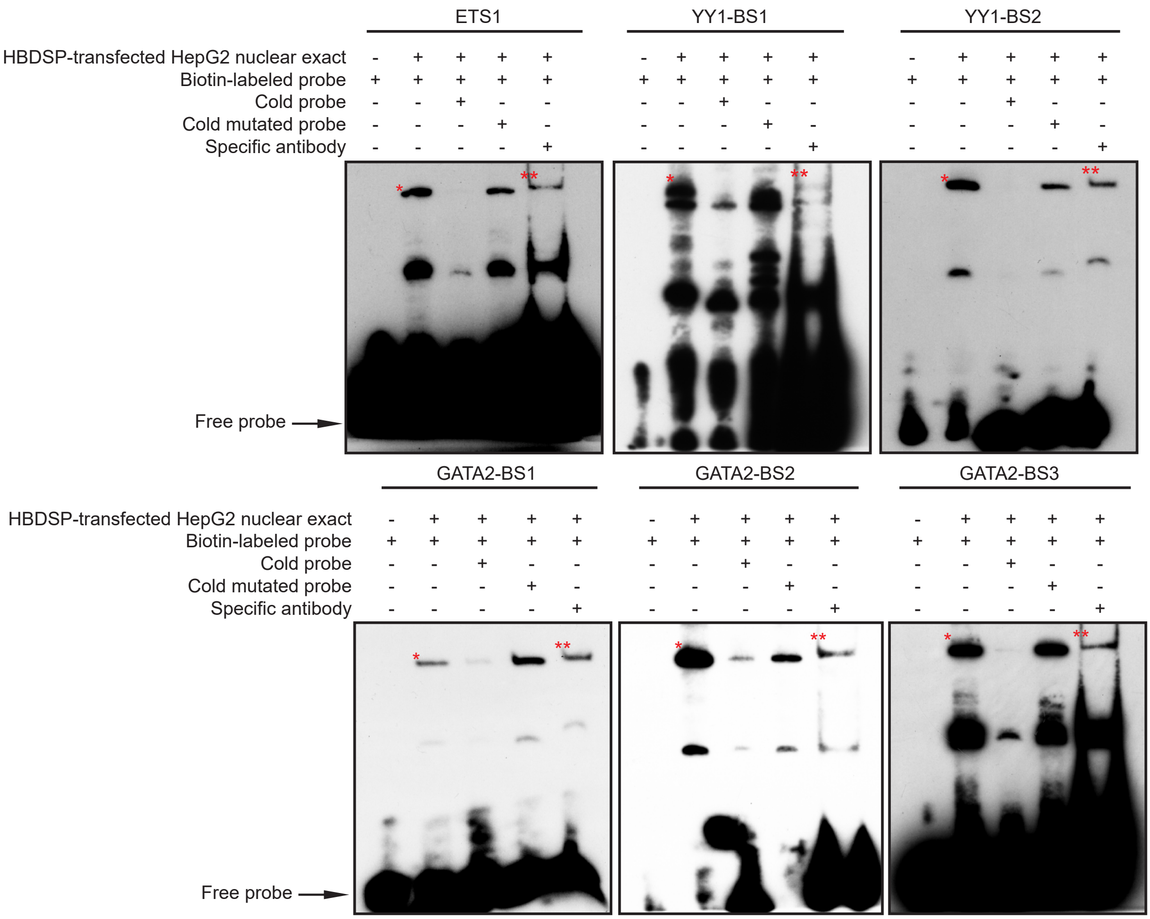
ETS1
YY1-BS1
YY1-BS2
HBDSP-transfected HepG2 nuclear exact
Biotin-labeled probe
Cold probe
Cold mutated probe
Specific antibody
Free probe
GATA2-BS1
GATA2-BS2
GATA2-BS3
HBDSP-transfected HepG2 nuclear exact
Biotin-labeled probe
Cold probe
Cold mutated probe
Specific antibody
Free probe

Fig S5

FIGURE S5. EMSA and super-shift assay of ETS1, GATA2, and YY1 binding to p53 promoter *in vitro* in HBDSP-transfected HepG2 cells. The 5'-biotin end-labeled probes were incubated in the absence (lane 1) or presence (lane 2) of nuclear extracts from HepG2 cells transfected with phouge-HBDSP. The unlabeled cold probes (lane 3) and unlabeled cold mutated probes (lane 4) were used as competitors at a concentration of 100-fold molar excess to the biotin-labeled probes. Super-shift assays were performed using 10 μg specific antibodies against ETS1 or GATA2 or YY1 (lane 5). Specific protein-DNA complexes (shown as shift band) are indicated as one asterisk, and the super-shift bands are marked by two asterisks. BS, Binding Site; * shift band; ** super-shift band.

Table S1 The wild-type and corresponding mutated sequences used for the identification of EST1, GATA2 and YY1 recognition sites

Plasmids	Recognition sites	Wild sequences (5'-3')	Mutated sequences (5'-3')
pGL4.10-p53-595-ETS1-mut	-463 ~ -460	TTCC	TTaa
pGL4.10-p53-595-GATA2-mut1	-535 ~ -531	TGATA	TaATA
pGL4.10-p53-595-GATA2-mut2	-421 ~ -417	TGATA	TaATA
pGL4.10-p53-595-GATA2-mut3	-385 ~ -381	TGATA	TaATA
pGL4.10-p53-595-YY1-mut1	-391 ~ -388	ATGG	tacc
pGL4.10-p53-595-YY1-mut2	-461 ~ -458	CCAT	tacc

The bases in lower case represent mutated EST1, GATA2 and YY1 recognition sites.

Table S2 Sequences of primers used in plasmid construction, qRT-PCR, ChIP-PCR and ChIP-qPCR

Plasmids	Primers (5'-3')	Assays
pGL4.10-p53-1509	F: 5'-GGGGTACCAGAGGTATCTTCCATGGCTT-3'	plasmid construction
	R: 5'-CCGCTCGAGACTCAGAGAGGACTCATCAA-3'	
pGL4.10-p53-1005	F: 5'-GGGGTACCCTGGGAGTAGGCAGAAGACT-3'	plasmid construction
	R: 5'-CCGCTCGAGACTCAGAGAGGACTCATCAA-3'	
pGL4.10-p53-497	F: 5'-GGGGTACCGTAGAAAAATTCTATATCCT-3'	plasmid construction
	R: 5'-CCGCTCGAGACTCAGAGAGGACTCATCAA-3'	
pGL4.10-p53-814	F: 5'-GGGGTACCGGACGTGAAAGGTTAGAAGG-3'	plasmid construction
	R: 5'-CCGCTCGAGACTCAGAGAGGACTCATCAA-3'	
pGL4.10-p53-595	F: 5'-GGGGTACCAGACCTGTCTCCCTCATTCA-3'	plasmid construction
	R: 5'-CCGCTCGAGACTCAGAGAGGACTCATCAA-3'	
pGL4.10-p53-360	F: 5'-GGGGTACCGTTAGGGTGTGATATTACGG-3'	plasmid construction
	R: 5'-CCGCTCGAGACTCAGAGAGGACTCATCAA-3'	
pGL4.10-p53-195	F: 5'-GGGGTACCAGCTCTGGCTTGCAGAATTT-3'	plasmid construction
	R: 5'-CCGCTCGAGACTCAGAGAGGACTCATCAA-3'	
pAcGFP-ETS1	F: 5'-GTCAGATCCGCTAGCGCCACCATGAGCTACTTTGTGGAT-3'	plasmid construction
	R: 5'-CGGGCCCGCGGTACCGTCTCGTCGGCATCTGGCTT-3'	
pAcGFP-GATA2	F: 5'-GTCAGATCCGCTAGCGCCACCATGGAGGTGGCGCCCGAG-3'	plasmid construction
	R: 5'-CGGGCCCGCGGTACCGTGCCCATGGCGGTCACCAT-3'	
pAcGFP-YY1	F: 5'-GTCAGATCCGCTAGCGCCACCATGGCCTCGGGCGACACC-3'	plasmid construction
	R: 5'-CGGGCCCGCGGTACCGTCTGGTTGTTTTTGGCCTT-3'	
p53	F: 5'-GCTGCTCAGATAGCGATGGT-3'	qRT-PCR
	R: 5'-ACAGTCAGAGCCAACCTCAG-3'	
GAPDH	F: 5'-CTCATGACCACAGTCCATGC-3'	qRT-PCR
	R: 5'-CAGTGAGCTTCCCGTTCAG-3'	
p53 (-595/-360)	F: 5'-AGACCTGTCTCCCTCATTCAAAAAA-3'	ChIP-PCR
	R: 5'-GTTTTCTCCCAGATACTTTATATCA-3'	
p5000	F: 5'-GTCTCGAACTCCTGACCTCAGGTGATCCAC-3'	ChIP-PCR
	R: 5'-GTTTATGGGTGGGCAAGAGTGAAATCCAGG-3'	
p53 (-595/-360)-Q	F: 5'-TAGGCACTCAGGAATACAACAATG-3'	ChIP-qPCR
	R: 5'-CCTTCACAACCCTTATCACTCTG-3'	

Underlined sequences represent the restriction enzyme recognition sites of vectors.

Table S3 Sequences of the siRNA oligo used in RNA interference

Gene symbols	Sequences (5'-3')
si-p53#1	sense: 5'-GACUCCAGUGGUAAUCUACTT-3'
	anti-sense: 5'-GUAGAUUACCACUGGAGUCTT-3'
si-p53#2	sense: 5'-GUAAUCUACUGGGACGGAATT-3'
	anti-sense: 5'-UUCCGUCCCAGUAGAUUACTT-3'
si-ETS1#1	sense: 5'-GCAGUUUCUUCUGGAAUUATT-3'
	anti-sense: 5'-UAAUUCCAGAAGAAACUGCTT-3'
si-ETS1#2	sense: 5'-GCACCUUCAAGGACUAUGUTT-3'
	anti-sense: 5'-ACAUAGUCCUUGAAGGUGCTT-3'
si-GATA2#1	sense: 5'-CACCUGUUGUGCAAAUUGUTT-3'
	anti-sense: 5'-ACAAUUUGCACAACAGGUGTT-3'
si-GATA2#2	sense: 5'-GCCUCUACCACAAGAUGAATT-3'
	anti-sense: 5'-UUCAUCUUGUGGUAGAGGCTT-3'
si-YY1#1	sense: 5'-CAGUCAACUAACCUGAAAUTT-3'
	anti-sense: 5'-AUUUCAGGUUAGUUGACUGTT-3'
si-YY1#2	sense: 5'-CUGGCAGAAUUUGCUAGAATT-3'
	anti-sense: 5'-UUCUAGCAAAUUCUGCCAGTT-3'

Table S4 Sequences of the probes used in EMSA

Probes	Sequences (5'-3')
ETS1-Biotin-labeled	F: 5'-AAGGCTTACGTTTCCATGTACTGAA-3'
	R: 5'-TTCAGTACATGGAAACGTAAGCCTT-3'
ETS1-Cold	F: 5'-AAGGCTTACGTTTCCATGTACTGAA-3'
	R: 5'-TTCAGTACATGGAAACGTAAGCCTT-3'
ETS1-Cold mutated	F: 5'-AAGGCTTACGTTTaaATGTACTGAA-3'
	R: 5'-TTCAGTACATTTAAACGTAAGCCTT-3'
GATA2-1-Biotin-labeled	F: 5'-TGCTACCCAGCACTGATATAGGCAC-3'
	R: 5'-GTGCCTATATCAGTGCTGGGTAGCA-3'
GATA2-1-Cold	F: 5'-TGCTACCCAGCACTGATATAGGCAC-3'
	R: 5'-GTGCCTATATCAGTGCTGGGTAGCA-3'
GATA2-1-Cold mutated	F: 5'-TGCTACCCAGCACTaATATAGGCAC-3'
	R: 5'-GTGCCTATATTAGTGCTGGGTAGCA-3'
GATA2-2-Biotin-labeled	F: 5'-AAATCTTATCAGAGTGATAAGGGTT-3'
	R: 5'-AACCCTTATCACTCTGATAAGATTT-3'
GATA2-2-Cold	F: 5'-AAATCTTATCAGAGTGATAAGGGTT-3'
	R: 5'-AACCCTTATCACTCTGATAAGATTT-3'
GATA2-2-Cold mutated	F: 5'-AAATCTTATCAGAGTaATAAGGGTT-3'
	R: 5'-AACCCTTATTACTCTGATAAGATTT-3'
GATA2-3-Biotin-labeled	F: 5'-AAATAAGATGGTGTGATATAAAGTA-3'
	R: 5'-TACTTTATATCACACCATCTTATTT-3'
GATA2-3-Cold	F: 5'-AAATAAGATGGTGTGATATAAAGTA-3'
	R: 5'-TACTTTATATCACACCATCTTATTT-3'
GATA2-3-Cold mutated	F: 5'-AAATAAGATGGTGTaATATAAAGTA-3'
	R: 5'-TACTTTATATTACACCATCTTATTT-3'
YY1-1-Biotin-labeled	F: 5'-AGGAGATTAAATAAGATGGTGTGAT-3'
	R: 5'-ATCACACCATCTTATTTAATCTCCT-3'
YY1-1-Cold	F: 5'-AGGAGATTAAATAAGATGGTGTGAT-3'
	R: 5'- ATCACACCATCTTATTTAATCTCCT-3'
YY1-1-Cold mutated	F: 5'-AGGAGATTAAATAAGtaccTGTGAT-3'
	R: 5'-ATCACAGGTACTTATTTAATCTCCT-3'
YY1-2-Biotin-labeled	F: 5'-AAGGCTTACGTTTCCATGTACTGAA-3'
	R: 5'-TTCAGTACATGGAAACGTAAGCCTT-3'
YY1-2-Cold	F: 5'-AAGGCTTACGTTTCCATGTACTGAA-3'
	R: 5'-TTCAGTACATGGAAACGTAAGCCTT-3'
YY1-2-Cold mutated	F: 5'-AAGGCTTACGTTTtaccGTACTGAA-3'
	R: 5'-TTCAGTACGGTAAAACGTAAGCCTT-3'

Underlined sequences represent the binding sites and lower-case sequences represent the mutated sites.
